# Ground truth labels challenge the validity of sepsis consensus definitions in critical illness

**DOI:** 10.1186/s12967-022-03228-7

**Published:** 2022-01-15

**Authors:** Holger A. Lindner, Shigehiko Schamoni, Thomas Kirschning, Corinna Worm, Bianka Hahn, Franz-Simon Centner, Jochen J. Schoettler, Michael Hagmann, Jörg Krebs, Dennis Mangold, Stephanie Nitsch, Stefan Riezler, Manfred Thiel, Verena Schneider-Lindner

**Affiliations:** 1 Department of Anesthesiology and Surgical Intensive Care Medicine, Medical Faculty Mannheim, Heidelberg University, Theodor-Kutzer-Ufer 1-3, 68167 Mannheim, Germany; 2 Mannheim Institute of Innate Immunoscience (MI3), Medical Faculty Mannheim, Heidelberg University, Theodor-Kutzer-Ufer 1-3, 68167 Mannheim, Germany; 3 Department of Computational Linguistics, Heidelberg University, Im Neuenheimer Feld 325, 69120 Heidelberg, Germany; 4 Interdisciplinary Center for Scientific Computing (IWR), Heidelberg University, INF 205, 69120 Heidelberg, Germany; 5 Department of Community Health Sciences, Max Rady College of Medicine, University of Manitoba, S113-750 Bannatyne Ave, Winnipeg, MB R3E 0W3 Canada

**Keywords:** Sepsis, Questionnaire survey, Ground truth, Expert label, SIRS, Suspicion of infection, Sepsis-1/2, Sepsis-3

## Abstract

**Background:**

Sepsis is the leading cause of death in the intensive care unit (ICU). Expediting its diagnosis, largely determined by clinical assessment, improves survival. Predictive and explanatory modelling of sepsis in the critically ill commonly bases both outcome definition and predictions on clinical criteria for consensus definitions of sepsis, leading to circularity. As a remedy, we collected ground truth labels for sepsis.

**Methods:**

In the Ground Truth for Sepsis Questionnaire (GTSQ), senior attending physicians in the ICU documented daily their opinion on each patient’s condition regarding sepsis as a five-category working diagnosis and nine related items. Working diagnosis groups were described and compared and their SOFA-scores analyzed with a generalized linear mixed model. Agreement and discriminatory performance measures for clinical criteria of sepsis and GTSQ labels as reference class were derived.

**Results:**

We analyzed 7291 questionnaires and 761 complete encounters from the first survey year. Editing rates for all items were > 90%, and responses were consistent with current understanding of critical illness pathophysiology, including sepsis pathogenesis. Interrater agreement for presence and absence of sepsis was almost perfect but only slight for suspected infection. ICU mortality was 19.5% in encounters with *SIRS* as the “worst” working diagnosis compared to 5.9% with *sepsis* and 5.9% with *severe sepsis* without differences in admission and maximum SOFA. Compared to *sepsis*, proportions of GTSQs with *SIRS* plus acute organ dysfunction were equal and macrocirculatory abnormalities higher (p < 0.0001). *SIRS* proportionally ranked above *sepsis* in daily assessment of illness severity (p < 0.0001)*.* Separate analyses of neurosurgical referrals revealed similar differences. Discriminatory performance of Sepsis-1/2 and Sepsis-3 compared to GTSQ labels was similar with sensitivities around 70% and specificities 92%. Essentially no difference between the prevalence of SIRS and SOFA ≥ 2 yielded sensitivities and specificities for detecting sepsis onset close to 55% and 83%, respectively.

**Conclusions:**

GTSQ labels are a valid measure of sepsis in the ICU. They reveal suspicion of infection as an unclear clinical concept and refute an illness severity hierarchy in the SIRS-sepsis-severe sepsis spectrum. Ground truth challenges the accuracy of Sepsis-1/2 and Sepsis-3 in detecting sepsis onset. It is an indispensable intermediate step towards advancing diagnosis and therapy in the ICU and, potentially, other health care settings.

**Supplementary Information:**

The online version contains supplementary material available at 10.1186/s12967-022-03228-7.

## Background

According the Global Burden of Diseases, Injuries, and Risk Factors Study, 20% of global deaths in 2017 were sepsis associated [1]. In the critically ill, global incidence and mortality of sepsis are both estimated around 30% [2, 3]. Delay of antibiotic treatment initiation in these patients is associated with increased mortality [4–9]. Considerable potential for improvement of sepsis outcomes thus lies in expediting its diagnosis. Yet, a gold standard test to establish the timely and definite diagnosis of sepsis and trigger initiation of antimicrobial therapy does not exist, and this treatment decision largely depends on the clinical assessment of a patient [10].

Broadly speaking, the term “sepsis” stands for the clinical hypothesis “about the nature of the patient’s problem”, verbatim from [11] (p. 10), that the host response to an infection is the primary cause of patient deterioration. A diagnosis of “sepsis” alerts to an immanently life-threatening condition that empirically requires rapid and specific therapy.

In the absence of a clear process understanding and a gold standard diagnostic test for sepsis, different concepts to define sepsis and clinical criteria to operationalize these concepts have been identified based on expert consensus. Thirty years ago, sepsis has first been defined as a disease syndrome with increasing severity from sepsis [systemic inflammatory response syndrome (SIRS) due to infection] to severe sepsis (sepsis with organ dysfunction) and septic shock (severe sepsis with hypotension despite adequate fluid resuscitation) [12]. In accordance to a presumed SIRS-sepsis hierarchy [13], critically ill patients with SIRS may, and those with sepsis are likely, to rapidly deteriorate into septic shock and multiple organ failure [14, 15]. This concept (Sepsis-1) provided the foundation for the development of clinical guidelines for sepsis management, in the course of which it was extended to include signs of altered homeostasis (Sepsis-1/2) [16, 17]. Recently, the Sepsis-3 definition has relegated the least severe sepsis category as per Sepsis-1/2 from the disease spectrum, deemed the term severe sepsis redundant, and conceptually defined sepsis as life-threatening organ dysfunction by a dysregulated host response to infection [18].

The need for better disease models and a pathomechanistic understanding of the sepsis syndrome persists [19–21]. Statistical analysis of comprehensive and complex intensive care unit (ICU) patient data from electronic health records (EHRs) holds promise to reveal new pathophysiological relationships [22, 23]. Above all, machine learning (ML) has the potential to advance sepsis prediction from static illness severity scoring to dynamic real-time early warning scores [22, 24, 25]. A reporting standard to retrospectively identify sepsis onset and severity in EHRs to this end is however lacking [26–29]. Manual chart review and administrative codes have been widely used instead [10, 30]. Various sets of clinical criteria to retrospectively implement existing definitions of sepsis and define sepsis onset in EHRs have been introduced [31, 32]. It is noteworthy that, in their original assessment of clinical criteria to support the Sepsis-3 definition, Seymour et al. [33] pointed out that their data did not mandate the evaluation of hospitalized patients with an elevated sequential organ failure assessment (SOFA) score at baseline for the presence of infection. Nevertheless, this strategy has become a mainstay for the identification of sepsis in EHRs from patients in the ICU and emergency department [32, 34–41].

The use of EHRs for research into predictive models to support timely sepsis diagnosis in the clinics requires pertinent labels for sepsis onset. Here, the moment when the diagnostic decision was actually made at the bedside is a self-evident initial choice. Clinical assessment in this situation is not exclusively determined by defined clinical criteria recorded in the EHR. Above all, physical examination of a patient is integral to clinical practice and a fundamental source of information for clinical thinking [11]. This thinking further critically draws upon different clinicians’ cognitive strategies including watchful waiting, theory of mind, heuristics, anticipatory thinking, and consultation [42, 43]. Those, who later attempt to retrace the clinical reasoning and decision-making process and retrospectively adjudicate the diagnosis, lack the personal experience of patient examination and strategic information gathering and processing. Notably, the agreement between sepsis diagnoses in consecutive, unselected patients made in the reality of clinical practice and sepsis onset defined retrospectively through surrogate strategies including Sepsis-1/2 and Sepsis-3 clinical criteria has not yet been evaluated.

Another crucial point when defining sepsis onset by clinical criteria in EHR data to develop predictive models consists in avoiding circular predictions. These occur when models contain clinical parameters that have already been used to define the outcome and thus essentially reconstruct the known deterministic functional definition of the target [44, 45]. Circularity limits the ability of a model to uncover yet unknown relationships in clinical data. This is exemplified, e.g., by studies that defined Sepsis-3 through the SOFA score and present ML models that predict Sepsis-3 based on the same clinical criteria that underlie SOFA [34, 35, 46].

We previously reported the use of implicit expert clinician knowledge, collected by daily electronic questionnaire survey among all patients in the ICU, for early sepsis detection by non-circular ML [44]. Here, we introduce the questionnaire that we named Ground Truth for Sepsis Questionnaire (GTSQ) and summarize results from the 1st year of the GTSQ survey in our interdisciplinary surgical ICU with a focus on the working diagnosis label for the presence of SIRS and sepsis. We separately analyzed neurosurgical patients as these represent a subgroup with distinct SIRS and sepsis etiologies [47, 48] commonly treated in our ICU. Since clinical reasoning exhibits inter-physician variability, we also evaluated interrater reliability for selected GTSQ items. Agreement and test performance of clinical criteria compared to GTSQ labels were evaluated. The significance of ground truth for advancing our understanding of sepsis is discussed.

## Methods

### Setting and data collection

The Department of Anesthesiology and Surgical Intensive Care Medicine at the University Medical Centre Mannheim, a 1352-bed tertiary care center operates a 22-bed interdisciplinary surgical ICU. The ICU houses six isolation room beds and a resuscitation area, and is a center in the Acute Respiratory Distress Syndrome Network Germany.

An electronic patient data management system (PDMS) (IntelliSpace Critical Care and Anesthesia, Philips, Eindhoven, the Netherlands) is used for all on-site monitoring and documentation including laboratory report server data. The PDMS data was linked with data from the hospital information system (HIS) (SAP, Walldorf, Germany) and the microbiology report server ([i/med], Dorner, Müllheim (Baden), Germany) forming our EHR. PDMS admissions were cross-validated using HIS data.

Two clinician scientists (F.S.C. and J.J.S.) determined 2 PM–2 PM SOFA scores [49] for all ICU patients by manual chart review as reported [50]. The ICU is run on a three-shift system. Senior intensivists were on call on a weekly basis and were responsible for editing the GTSQ during this period daily at 2 PM for all ICU patients.

### GTSQ structure and content

The ten questionnaire items are numbered by their order in the final GTSQ version (Table 1 and Additional file 1: Appendix S1). They were intended to capture current expert opinions. The experts were not provided with any written or verbal instructions for answering the survey, particularly for assigning the working diagnosis. As contextual information, Item 1 requested assigning each patient to the three most or three least severely ill of all concurrently treated ICU patients or to none of these two groups. It was not intended to reflect resource consumption in care. The remaining nine items covered the three sepsis-related domains diagnosis (Items 3–5 and 7–9), intervention (Item 6), and outcome (Items 2 and 10). Item 3 identified one of five working diagnoses, italicized in the following, that are presumably associated with increasing severity of illness: (1) *neither SIRS nor sepsis*, (2) *SIRS*, (3) *sepsis*, (4) *severe sepsis*, or (5) *septic shock*. Item 4 assessed suspicion of infection and Item 5 mapped a suspected or confirmed focus of infection to a list of nine possible localizations. Item 7 evaluated macrocirculatory abnormalities and Item 8 suspicion of microcirculatory dysfunction. Item 9 captured dysfunction for eight different organs and classified it into acute, which was mainly considered in this study, and chronic pre-existing. Additionally, the cause of organ dysfunctions was classified into infectious, non-infectious and unclear. Item 6 recorded source control measures. Item 2 judged the trend in the overall clinical picture for the preceding 24 h and Item 10 the expected development of patient status during the next 24 h, each classified into improvement, no change or deterioration. In addition to pre-specified reply options, Items 2, 4, 6 and 10 allowed free text entries. Additional file 1: Text S1 provides a description of GTSQ development and survey implementation. For the survey, the GTSQ was transferred to a tablet PC.

**Table 1:** Quick reference table for GTSQ items

Item	Topic
1	Daily rating of illness severity
2	Patient’s development in last 24 h
3	Working diagnosis
4	Suspicion of infection
5	Focus of infection
6	Source control
7	Macrocirculatory abnormalities
8	Microcirculatory dysfunctions
9	Acute organ dysfunction
10	Expected 24-h trend

### Study population and definitions

**Fig. 1:**
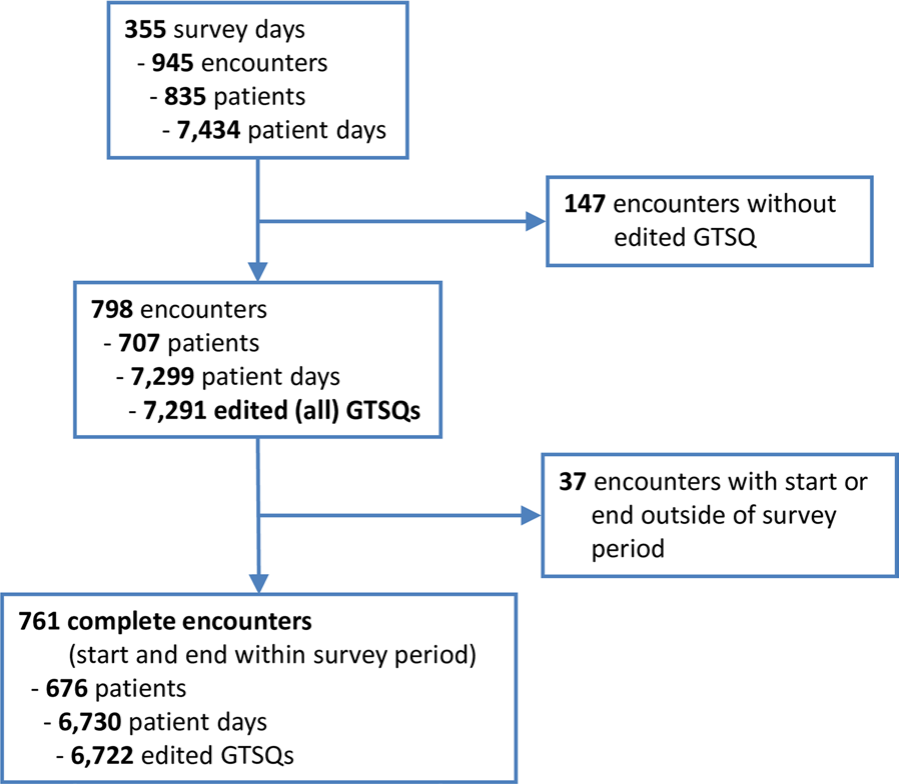
Flow diagram of Ground Truth for Sepsis Questionnaire (GTSQ) survey

We counted 962 ICU admissions ongoing or beginning during the survey period, from which we derived 945 encounters as described in Additional file 1: Text S2. We included all encounters with at least one edited questionnaire, no other exclusion criteria were applied (Fig. 1). This way, the study population can be considered as an unselected cohort of consecutive admissions to our ICU. We defined start and end of encounters minimizing time that these patients were putatively not under direct observation by our raters as detailed in Additional file 1: Text S2. Briefly, PDMS admission times more closely matched the first and HIS discharge times the last charting time for either peripheral oxygen saturation or heart rate, herein referred to as vital signs. Therefore, the time of PDMS admission or the first vital sign, whichever was first, marked the encounter start, and the time of HIS discharge or the last vital sign, whichever was last, marked the encounter end. These time points thus defined patient time.

GTSQs with at least one reply to any of the Items 2–10, including free text entries, were considered edited. Contextual Item 1 was excluded here because the assignment to neither the three most nor the three least severely ill patients was the default setting and was thus implicit if unchanged. The proportion of encounters captured by GTSQ-rating in a given time span is referred to as GTSQ-editing rate.

“On-admission” refers to the documentation of a given characteristic, e.g., working diagnosis label (Item 3) or SOFA score, already on the first 2 PM-rating time point after the start of an encounter, i.e., on day 1. In the absence of documentation on day 1, day 2 was still considered as on-admission. The highest SOFA score during an encounter was defined as “maximum SOFA”.

### Data analysis

GTSQ-data collected from 18/07/2016–08/07/2017 was linked to our EHR. Missing replies to items were treated as missing data. Antimicrobial therapy was extracted from the PDMS and microbiology testing from microbiology report data.

We analyzed survey responses and clinical characteristics at the level of the complete encounters (encounter level) and at the individual questionnaire level (GTSQ level). Groups were generally compared with t-tests, except for SOFA scores, for which we used Mann–Whitney-U tests, and proportions were compared with chi squared tests. Comparisons at the GTSQ level may underestimate variance because of stronger intra-individual than inter-individual correlations between GTSQ results. We therefore compared SOFA scores between working diagnosis groups with a generalized linear mixed model to consider that the scores were clustered by encounter and had a temporal sequence. The model consisted of the SOFA score as outcome (response or dependent variable) and the five working diagnoses categories as predictor (independent variable). No other covariates, non-linear terms, or interactions were included as fixed effects. We considered as random effects (1) the encounter identifier (subject-specific intercept) and (2) an exponential temporal covariance structure for the correlation between SOFA scores of the same patient (option type = SP(POW) in the random statement of the SAS procedure GLIMMIX). The resulting p-values from multiple group comparisons were Bonferroni-adjusted.

We used SAS V9.4 for data analysis and considered p-values of < 0.05 as statistically significant.

### Interrater reliability

We conducted an interrater reliability analysis on 126 patients for rating the working diagnosis (Item 3), suspicion of infection (Item 4), macrocirculatory abnormalities (Item 7), acute and chronic pre-existing organ dysfunction (Item 9), and expected 24-h trend (Item 10). Between 29/03/2017 and 14/06/2017, three of our raters each additionally edited a printed questionnaire version for these five items on 2 non-consecutive days so that all three possible rater pairs rated the items twice concomitantly. We calculated Krippendorff’s α (K_α_) as principal measure of agreement and provide prevalence-adjusted bias-adjusted kappa [51] and other measures where appropriate (see Additional file 1: Text S3 for further details).

### Automated sepsis detection by clinical criteria and agreement with ground truth labels

In accordance with Seymour et al. [33], we determined the onset of infection, also referred to as suspicion of infection, in complete encounters by automated retrieval of orders for administration of antibiotics and body fluid culture. As described [33], a 72-h infection window was defined starting 48 h before and ending 24 h after the moment of suspicion of infection. Data to determine the presence of SIRS and the SOFA score was extracted from the PDMS (Additional file 1: Text S4). For all variables, a data entry was carried forward until replaced by a new entry. The presence of SIRS and the SOFA score were updated at every instant a new data entry became available or at least every 30 min. SIRS was present if two or more of the four SIRS criteria were met concomitantly [12]. Thereby, SIRS was not determined as a summary measure for a given time interval but as a point determination as originally devised [12]. The SOFA score, by contrast, was calculated as the sum of the highest value of each of its six sub-scores within the preceding 24 h as originally devised [49]. The Sepsis-1/2 clinical criteria were considered met at the onset of infection if, at any time in the infection window, SIRS was present and Sepsis-3 if the SOFA score reached a value ≥ 2. Alternatively, Sepsis-3 was considered present if the SOFA score increased by two points or more at any time in the infection window.

Agreement and test performance of clinical criteria were evaluated as indicated with GTSQ labels (Item 3) as reference class. In 51 encounters with on-admission sepsis but no working diagnosis label on day 1, the specific sepsis label given on day 2 was transferred to the first 2 PM-rating time point. Bedsides the percent agreement, K_α_ is reported as measure of agreement, sensitivity and specificity as well as positive and negative predictive values (PPV and NPV) as measures of test performance, and additionally the positive and negative class agreements as indicated.

### Glossary

As a quick reference, the following glossary is intended to support the reading flow.

#### Encounter

An *encounter* covers an episode of a hospital stay, during which a patient was not only administratively filed as an intensive care patient but was actually treated by our senior intensivists and was thus expected to be included into the GTSQ survey. The term *all encounters* refers to the total of 798 encounters included in the study (cf. Fig. 1). Among these, 761 so-called *complete encounters* started and ended within the survey period. Analyses at the *encounter level* considered all 7291 edited GTSQs from all 798 encounters.

#### GTSQ

The acronym *GTSQ* stands for *G*round *T*ruth for *S*epsis *Q*uestionnaire. The ten-item questionnaire captures and further characterizes the patient condition referred to as “sepsis” according to the current opinion of the treating senior intensivist at the bedside. In the *GTSQ survey*, senior intensivists rated every individual ICU patient daily at 2 PM, thereby, creating individual *edited GTSQs*. The terms *GTSQ label*, *ground truth label* and *expert label* are used interchangeably and refer to the reply for a given questionnaire item.

#### On-admission

*On-admission* indicates that a given GTSQ label or the SOFA score was available by no later than the second 2 PM-rating time point of an encounter.

#### Referral group

Encounters referred to the ICU by a given clinical department or group of departments constitute a *referral group*. In our subgroup analysis, encounters are accordingly divided into a neurosurgical and non-neurosurgical referral group.

#### Suspicion of infection

*Suspicion of infection* refers to the hypothesis of invasion by a pathogen. As a clinical suspicion, it is captured by GTSQ Item 4. Consensus definitions of sepsis commonly resort to temporal connections between antimicrobial therapy and microbiology testing as clinical criteria to establish *suspicion of infection* in EHRs.

## Results

### The GTSQ survey

During the 355-day survey period, there were 945 unselected encounters with 7434 patient days in our ICU (Fig. 1). We excluded 147 encounters without any edited questionnaire. Of these, 133 (90.5%) lasted less than 24 h. For the remaining 798 encounters, 150 (18.8%) of which lasted less than 24 h, 7291 questionnaires (median 5, range 1–103) were edited corresponding to 92.1% of the cumulative number of 2 PM-rating time points in these encounters, i.e., the editing rate. These are henceforward referred to as all encounters and all edited GTSQs (or all GTSQs), respectively.

Among all encounters, 761 started and ended within the survey period. These are referred to as complete encounters and contributed 6722 edited GTSQs (median 4, range 1–103). Of these, 85 were re-encounters. Median patient times in the first and last 2 PM–2 PM-rating intervals of complete encounters were 17.0 h (range 0.03–24 h) and 19.8 h (range 0.02–24 h), respectively. For 192 complete encounters (25.2%), first and last intervals were identical. The GTSQ-editing rates were 77.8% for first and 52.7%, for last rating intervals compared to 98.6% for all intervening intervals together that were from 536 encounters (70.4%).

Patients were ranked according to Item 1 on 350 out of 355 survey days. Otherwise, response rates for all GTSQs ranged from 90.6% for Item 4 (suspicion of infection) to 98.4% for Item 3 (working diagnosis). SOFA scores were available for 99.3% of all GTSQs. For all encounters, vital signs were charted at intervals ≤ 24 h for 98.2% of total patient time. For the complete encounters, vital signs were charted regularly in 98.4% of the patient time.

Overall, the completeness of our GTSQ survey and availability of concurrent clinical information together provide a basis for joint analyses in these data sources.

### Interrater reliability

Overall agreement for the five working diagnoses was almost perfect [52] when considered as ordinal and in a two-rater setting [K_α_ = 0.94 (95% CI 0.90–0.97)] (Additional file 1: Tables S1, S2 and Text S5). Disagreements were rare and of smaller magnitude, as indicated by the high K_α_ for the ordinal consideration. The lowest K_α_ = 0.70 for the *sepsis* category may be due to the very low prevalence of *sepsis* in relation to the other diagnoses which reduces kappa [51], nonetheless it still represents substantial agreement. The binary discrimination between septic and non-septic states also yielded very high values for the two-rater setting [K_α_ = 0.94 (95% CI 0.86–1.0)].

Agreement was substantial for both macrocirculatory abnormalities [K_α_ = 0.77 (95% CI 0.63–0.90)] and organ dysfunction [K_α_ = 0.68 (95% CI 0.43–0.88)], but only fair for the expected 24-h trend, not further discussed here, and slight for suspected infection (Additional file 1: Text S5 and Table S2). Additional file 1: Table S2 summarizes all K_α_ values for the interrater reliability study, and further agreement measures are provided in Additional File 1: Table S3.

### Encounter level

#### Working diagnoses and patient characteristics

The working diagnosis (Item 3) is the central ground truth label in this report. It was assigned at least once in 757 of 761 complete encounters. On only 2 days of the 355-day survey period, no patient was assigned any working diagnosis, on 2 days only 2 patients were rated (each as *septic shock*), and a working diagnosis was missing for 5, 4 and 3 patients on 2 days each, for 2 patients on 3 days, and for a single patient on 21 days. On 321 days, there was no missing rating for this item.

In 732 complete encounters, the first working diagnosis was available for day 1 or 2, i.e., on-admission. It was *neither SIRS nor sepsis* in 409 encounters, *SIRS* in 118, *sepsis* in 27, *severe sepsis* in 31, and *septic shock* in 147. A label for s*epsis*, *severe sepsis*, and* septic shock* was preceded by a non-sepsis label in 40, 13, and 56 of the 732 complete encounters, respectively, and 18 were neither definitely on-admission nor incident sepsis cases.

Thus, in 205 (26.9%) of all complete encounters, a sepsis label was present on admission, and 109 (14.3%) were incident sepsis cases. Encounters with sepsis on admission were mostly referrals from general surgery (44.4%) followed by anesthesiology (17.6%) while incident sepsis developed primarily in neurosurgical referrals (51.4%) followed by general surgery (21.1%). Mortality was about two times higher for encounters with sepsis on admission (37.6%) than for incident sepsis (18.4%) (p = 0.0005).

**Fig. 2:**
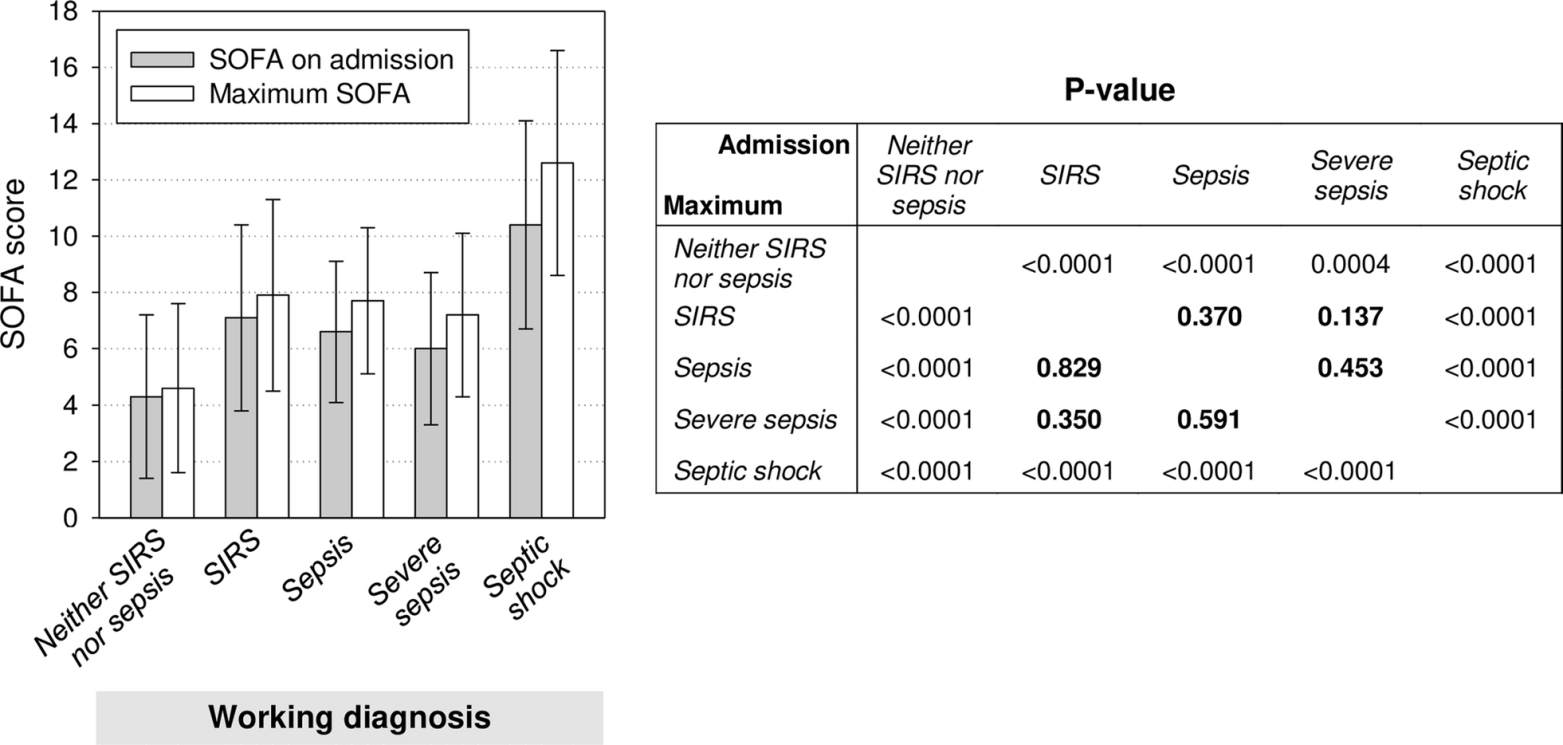
SOFA scores for complete encounters by working diagnosis (Item 3). SOFA scores on admission (gray) and maximum SOFA scores during ICU treatment (white) are represented as mean values (bars) with standard deviations (whiskers). The table shows p-values from the Mann–Whitney-U test for all between-working diagnosis differences in on-admission and maximum SOFA scores, respectively, above and below the diagonal, p-values from t-tests were consistent. The absence of statistical significance is highlighted by bold print of p-values

Considering the presumably most severe working diagnosis during an encounter as an absorbing state, 41.0% of the 761 complete encounters were categorized as *neither SIRS nor sepsis*, 14.8% as *SIRS*, 6.7% as *sepsis*, 4.5% as *severe sepsis*, and 32.5% as *septic shock* (Table 2). We applied this categorization in the following comparisons of complete encounters. The proportion of men in the three sepsis categories together was 66.3% compared to 56.2% in the two non-sepsis categories (p = 0.005). Age group distributions were similar among all working diagnosis categories. Neurosurgery was the referring department most strongly represented overall and in all single working diagnosis categories but *septic shock* (see subgroup analysis below). General surgery was second overall and first in *septic shock*. Internal medicine accounted for < 2% of all referrals. ICU mortalities were similarly low in the three categories *neither SIRS nor sepsis* (6.4%), *sepsis* (5.9%), and *severe sepsis* (5.9%), almost equal to overall mortality (19.2%) in the *SIRS* category (19.5%) and twice as high in the *septic shock* category (39.7%). For encounters in the combined sepsis categories, the Charlson comorbidity index (CCI), microbiology testing and antimicrobial treatment frequency were higher and ICU stay was longer than in the non-sepsis categories (p ≤ 0.0001 for each comparison). SOFA scores on admission and encounter maxima were highest in the *septic shock* category and lowest in *neither SIRS nor sepsis* (Fig. 2). Only in these two extreme categories, mean SOFA scores were significantly different from all other categories. Otherwise, we interpret the absence of statistical significance as absence of a clinically relevant difference.

**Table 2:** Characteristics of complete encounters in the survey period by working diagnosis (Item 3)

Characteristic	All (n = 761)^a^	Working diagnosis (Item 3)^b^				
		Neither SIRS nor sepsis	SIRS	Sepsis	Severe sepsis	Septic shock
		(n = 312)	(n = 113)	(n = 51)	(n = 34)	(n = 247)
Demographics						
Men	460 (60.4)	170 (54.5)	69 (61.1)	39 (76.5)	22 (64.7)	159 (64.4)
Age in yr mean (sd)	61.9 (16.2)	61.6 (16.3)	60.4 (18.2)	65.7 (15.8)	57.3 (19.7)	62.8 (14.6)
< 40 yr	80 (10.5)	38 (12.2)	16 (14.2)	3 (5.88)	5 (14.7)	18 (7.3)
40–60 yr	252 (33.1)	100 (32.1)	41 (36.3)	13 (25.5)	13 (38.2)	84 (34.0)
> 60 yr	429 (56.4)	174 (55.8)	56 (49.6)	35 (68.6)	16 (47.1)	145 (58.7)
Referring department^c^						
Anesthesiology	42 (5.5)	1 (0.3)	1 (0.9)	–	2 (5.9)	38 (15.4)
General surgery	168 (22.1)	19 (6.1)	29 (25.7)	13 (25.5)	6 (17.6)	100 (40.5)
Neurosurgery	364 (47.8)	215 (68.9)	57 (50.4)	19 (37.3)	15 (44.1)	57 (23.1)
Orthopedics and trauma center	87 (11.4)	33 (10.6)	12 (10.6)	10 (19.6)	5 (14.7)	26 (10.5)
Otorhinolaryngology	34 (4.5)	12 (3.9)	4 (3.5)	4 (7.8)	3 (8.8)	11 (4.5)
Urology	27 (3.6)	10 (3.2)	6 (5.3)	4 (7.8)	1 (2.9)	6 (2.4)
Other	43 (5.6)	24 (7.6)	5 (4.4)	1 (2.0)	2 (5.9)	10 (4.0)
Working diagnosis present						
On admission^d^	–	308 (98.7)	74 (65.5)	25 (49.0)	19 (55.9)	161 (65.2)
Charlson comorbidity index						
Mean (sd)	2.9 (2.8)	2.6 (2.9)	2.5 (2.4)	3.3 (3.2)	3.4 (3.0)	3.4 (2.6)
SOFA^e^, score value						
On admission						
Mean (sd)	6.9 (4.2)	4.3 (2.9)	7.1 (3.3)	6.6 (2.5)	6.0 (2.7)	10.4 (3.7)
Median (range)	6 (0–21)	4 (0–16)	7 (0–16)	7 (1–12)	6.5 (0–11)	10 (1–21)
Maximum						
Mean (sd)	8.0 (4.8)	4.6 (3.0)	7.9 (3.4)	7.7 (2.6)	7.2 (2.9)	12.6 (4.0)
Median (range)	7.5 (0–23)	4 (0–16)	8 (0–16)	8 (2–13)	7.5 (0–12)	12 (2–23)
Microbiology testing						
Blood cultures						
Mean (sd)	3.8 (6.1)	0.8 (1.4)	2.3 (2.7)	4.4 (5.1)	5.4 (4.9)	8.0 (8.4)
Median (range)	1 (0–48)	0 (0–9)	1 (0–15)	3 (0–23)	4 (0–21)	5 (0–48)
Bronchial lavage						
Mean (sd)	1.1 (3.0)	0.1 (0.4)	0.5 (1.2)	0.9 (1.6)	1.2 (1.8)	2.7 (4.7)
Median (range)	0 (0–50)	0 (0–3)	0 (0–7)	0 (0–6)	0 (0–9)	1 (0–50)
Antimicrobial therapy, ddd						
Mean (sd)	12.1 (31.3)	0.5 (2.4)	1.2 (5.0)	6.9 (9.4)	13.0 (18.8)	32.9 (47.6)
Median (range)	0.0 (0.0–421.8)	0.0 (0.0–23.4)	0.0 (0.0–45.0)	4.6 (0.0–46.5)	7.3 (0.0–99.1)	17.5 (0.0–421.8)
Length of encounter, d						
Mean (sd)	8.8 (11.9)	3.1 (3.6)	5.6 (5.3)	9.3 (8.6)	11.8 (8.8)	17.2 (16.1)
Median (range)	4.5 (0.1–104.9)	1.6 (0.2–20.4)	3.8 (0.3–22.5)	6.8 (0.6–33.7)	9.6 (0.4–33.8)	12.7 (0.1–104.9)
ICU mortality	146 (19.2)	20 (6.4)	22 (19.5)	3 (5.9)	2 (5.9)	98 (39.7)

*sd* standard deviation, *ddd* defined daily dose

^a^Data represent absolute numbers (% of column category) unless otherwise indicated

^b^Most severe working diagnosis assigned during encounter (*neither SIRS nor sepsis* > *SIRS* > *severe sepsis* > *septic shock*). The working diagnosis was missing for four complete encounters (0.5%)

^c^For 8 encounters, more than one referring department was listed. For 5 encounters, no referring department was documented. Of these, 4 were from the *neither SIRS nor sepsis* and 1 was from the *SIRS* working diagnosis category

^d^Working diagnosis labels were missing for both day 1 and 2 in 4 encounters in the *neither SIRS nor sepsis*, 3 in the *SIRS*, 3 in the *sepsis*, 1 in the *severe sepsis*, and 14 in the *septic shock* category

^e^SOFA scores at admission were determined at the first 2 PM-rating time point of each encounter and were available for all but two encounters, one of which had ended before the next rating time point

**Fig. 3:**
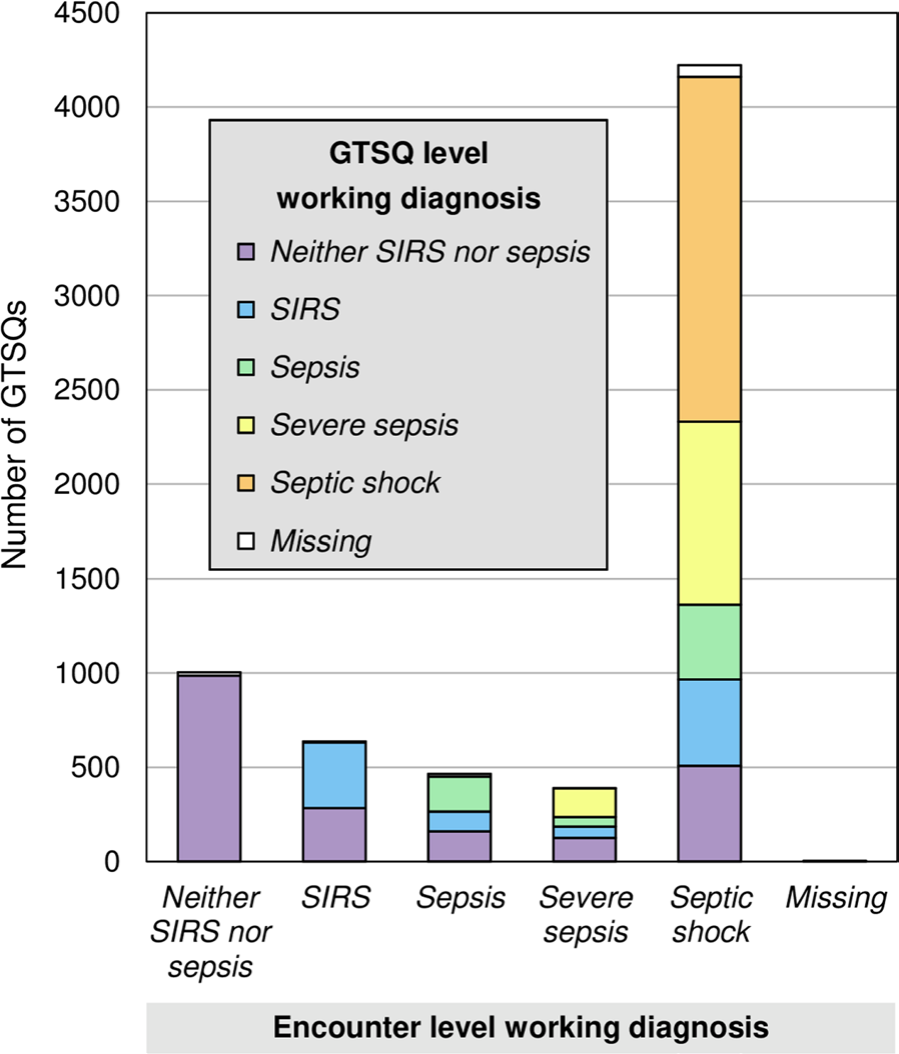
Working diagnosis label distribution in GTSQs from complete encounters by encounter working diagnosis (Item 3). Data include missing labels and is represented as stacked bar chart

Figure 3 displays the working diagnosis label distribution in the GTSQs from the complete encounters by encounter working diagnosis category. Among the 6722 edited GTSQs, 45.1% carried a non-sepsis and 53.3% a sepsis working diagnosis label. The GTSQs with a non-sepsis label were similarly divided across the sepsis-free encounters (53.3%) and the sepsis encounters (46.7%).

The more than threefold higher mortality rate for encounters in the *SIRS* compared to the *sepsis* and *severe sepsis* categories is conspicuous. The same applies to the absence of statistically significant differences in admission and maximum SOFA score across these three categories. These observations do not support a generally higher illness severity in sepsis compared to SIRS.

#### Course of illness

**Fig. 4:**
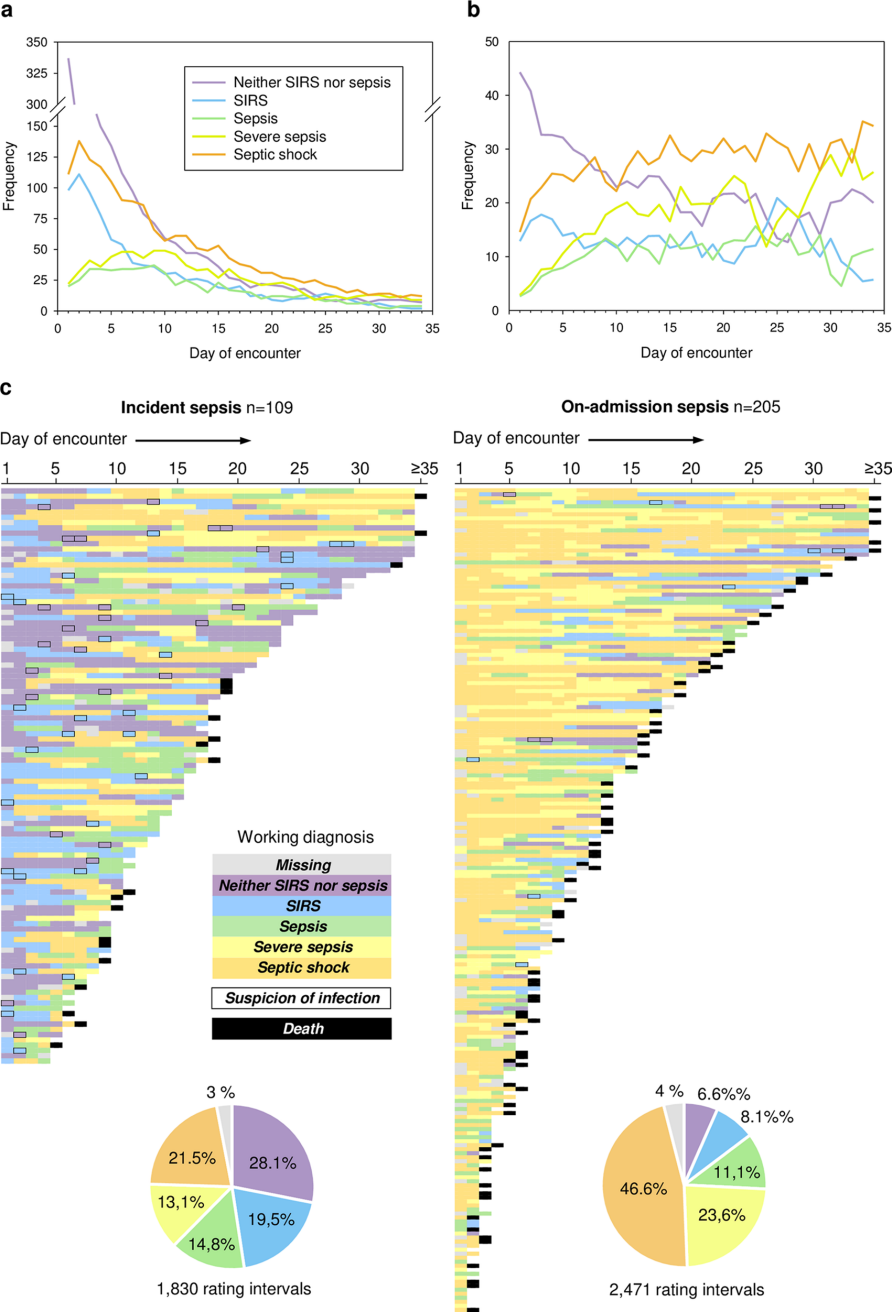
Distribution of working diagnosis labels (Item 3) during 34 days of follow-up for complete encounters. **a** Label frequencies and **b** prevalence. **c** Color map representation of label sequences and suspicion of infection for 109 incident (left) and 205 present-on-admission (right) sepsis cases. Encounters are sorted by descending length. For each day of follow-up, a rectangle colored as indicated in the legend identifies the working diagnosis (Item 3) and concurrent suspicion of infection with a non-sepsis label (Item 4). A black rectangular subsequent to the last expected rating indicates death in the ICU during this or the next rating interval, or beyond day 34

Patient stability in critical illness varies. Therefore, we sought a first cursory description of the disease course based on the working diagnosis labels (Item 3). This does not yet represent an analysis of time dependencies. GTSQs from the 761 complete encounters were grouped by consecutive encounter days and temporal distributions of working diagnosis labels were analyzed. During the first 34 encounter days, absolute counts of non-sepsis and *septic shock* labels decreased steadily, while *sepsis* and *severe sepsis* increased for up to 9 days before declining again (Fig. 4a). The proportion of the *neither SIRS nor sepsis* label declined in favor of all sepsis labels (Fig. 4b).

In our 109 incident sepsis cases, almost half of the ratings in this period featured a non-sepsis and half a sepsis label compared to 14.7% non-sepsis and 81.3% sepsis labels in our 205 present-on-admission sepsis cases (Fig. 4c). The median time to an incident sepsis label was 6 rating days (range 2–20).

**Fig. 5:**
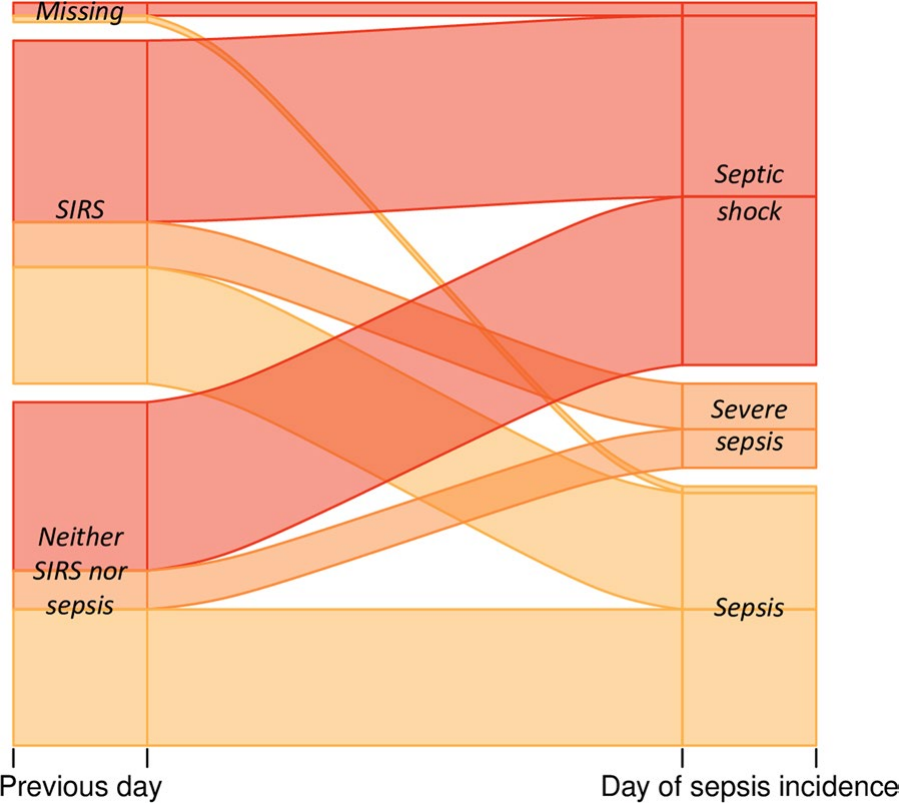
Alluvial plots for day-to-day working diagnosis transitions (Item 3). The day of the first sepsis diagnosis label for all 109 incident sepsis cases in complete encounters is shown on the right and labels for the preceding day on the left

For the 109 incident sepsis cases, the working diagnosis label immediately preceding the first sepsis label was available 106 times (Fig. 5). It was 53 times *neither SIRS nor sepsis* as well as 53 times *SIRS*. From both these labels, very similar numbers, respectively, transitioned into *sepsis* (21 and 18), *severe sepsis* (6 and 7), and *septic shock* (26 and 28) on the following day.

### GTSQ level

#### Working diagnoses and clinical characteristics

Most of the 7291 edited GTSQs had a working diagnosis label of *neither SIRS nor sepsis* (30.3%) or *septic shock* (27.6%) followed by *severe sepsis* (16.0%), *SIRS* (14.5%), and *sepsis* (10%) (Table 3). The median numbers and interquartile ranges of GTSQs per contributing encounter slightly increased from *neither SIRS nor sepsis* to *septic shock* but were not different for *SIRS* compared to *severe sepsis* suggesting little or no length bias in the following comparisons.

**Table 3:** Summary of survey results and frequencies for microbiology testing and antimicrobial use for all GTSQs by working diagnosis (Item 3)

Item (number) or characteristic	Affirmative/non-affirmative responses^a^	Affirmative responses^b^				
	All edited GTSQs (n = 7291)	Neither SIRS nor sepsis (n = 2206)	SIRS (n = 1056)	Sepsis (n = 732)	Severe sepsis (n = 1167)	Septic shock (n = 2012)
Encounters contributing n GTSQs	798	523	266	183	174	264
GTSQs per contributing encounter						
Median (interquartile range)	5 (2–12)	2 (1–6)	3 (2–5)	3 (2–5)	4 (2–9)	5 (2–10)
Suspected infection (4)	376/6232 (90.6)	70 (3.2)	87 (8.2)	56 (7.7)	48 (4.1)	115 (5.7)
Focus localization (5)	4184/2982 (98.3)	233 (10.6)	232 (22.0)	646 (88.3)	1124 (96.3)	1949 (96.9)
Localization unclear	153 (2.1)	45 (2.0)	30 (2.8)	18 (2.5)	9 (0.8)	51 (2.5)
Abdominal, suspected	387 (5.3)	9 (0.4)	21 (2.0)	33 (4.5)	91 (7.8)	233 (11.6)
Abdominal, confirmed	1390 (19.1)	23 (1.0)	38 (3.6)	150 (20.5)	454 (38.9)	725 (36.0)
Thoracic, suspected	834 (11.4)	23 (1.0)	48 (4.6)	124 (16.9)	199 (17.1)	440 (21.9)
Thoracic, confirmed	1381 (18.9)	68 (3.1)	69 (6.5)	158 (21.6)	432 (37.0)	654 (32.5)
Urogenital, suspected	49 (0.7)	2 (0.1)	4 (0.4)	5 (0.7)	16 (1.4)	22 (1.1)
Urogenital, confirmed	110 (1.5)	2 (0.1)	1 (0.1)	22 (3.0)	35 (3.0)	50 (2.5)
Intracranial/meningeal, suspected	35 (0.5)	2 (0.1)	5 (0.5)	8 (1.1)	3 (0.3)	17 (0.8)
Intracranial/meningeal, confirmed	239 (3.3)	34 (1.5)	5 (0.5)	58 (7.9)	68 (5.8)	74 (3.7)
Bone/joint, suspected	65 (0.9)	9 (0.4)	4 (0.4)	7 (1.0)	22 (1.9)	23 (1.1)
Bone/joint, confirmed	336 (4.6)	22 (1.0)	13 (1.2)	59 (8.0)	52 (4.5)	190 (9.4)
Skin, suspected	106 (1.5)	2 (0.1)	1 (0.1)	26 (3.6)	44 (3.8)	33 (1.6)
Skin, confirmed	188 (2.6)	8 (0.4)	2 (0.2)	38 (5.2)	29 (2.5)	111 (5.5)
Blood stream, suspected	30 (0.4)	1 (0.1)	–	4 (0.6)	5 (0.4)	20 (1.0)
Blood stream, confirmed	243 (3.3)	11 (0.5)	7 (0.7)	61 (8.3)	62 (5.3)	102 (5.1)
Catheter, suspected	27 (0.4)	1 (0.1)	–	8 (1.1)	1 (0.1)	17 (0.8)
Catheter, confirmed	10 (0.1)	1 (0.1)	–	4 (0.6)	–	5 (0.3)
Endocarditis^c^, suspected	6 (0.1)	1 (0.1)	1 (0.1)	1 (0.1)	3 (0.3)	–
Endocarditis, confirmed	6 (0.1)	–	–	3 (0.4)	–	3 (0.2)
More than 1 focus of infection	1242 (17.0)	23 (1.0)	17 (1.6)	128 (17.5)	352 (30.2)	722 (35.9)
Source control (6)	432/6,647 (97.1)	22 (1.0)	10 (1.0)	61 (8.3)	52 (4.5)	284 (14.1)
Surgical	354 (4.9)	18 (0.8)	6 (0.6)	41 (5.6)	39 (3.3)	247 (12.3)
Interventional	43 (0.6)	–	2 (0.2)	7 (1.0)	11 (0.9)	23 (1.1)
Catheter change	35 (0.5)	4 (0.2)	–	11 (1.5)	2 (0.2)	18 (0.9)
Other	15 (0.2)	–	2 (0.2)	4 (1.6)	1 (0.1)	8 (0.4)
Acute organ dysfunction (9)^d^	5267/1649 (94.9)	963 (43.7)	756 (71.6)	524 (71.6)	1086 (93.1)	1938 (96,3)
Gastrointestinal	831 (11.4)	41 (1.86)	8 (6.44)	69 (9.43)	176 (15.1)	477 (23.7)
Lung	3961 (54.3)	348 (15.8)	511 (48.4)	372 (50.8)	948 (81.2)	1782 (88.6)
Kidney	2266 (31.1)	130 (5.89)	236 (22.3)	109 (14.9)	567 (48.6)	1224 (60.8)
Brain	1796 (24.6)	628 (28.5)	292 (27.7)	160 (21.9)	204 (17.5)	512 (25.4)
Heart	1061 (14.6)	58 (2.63)	125 (11.8)	57 (7.79)	118 (10.1)	703 (34.9)
Coagulation system	730 (10.0)	29 (1.31)	51 (4.83)	38 (5.19)	124 (10.6)	488 (24.3)
Bone marrow	973 (13.3)	34 (1.54)	93 (8.81)	47 (6.42)	203 (17.4)	596 (29.6)
Liver	805 (11.0)	13 (0.59)	71 (6.72)	15 (2.05)	168 (14.4)	538 (26.7)
Macrocirculatory abnormalities (7)	3060/4008 (96.9)	256 (11.6)	402 (38.1)	162 (22.1)	332 (28.4)	1907 (94.8)
Volume replacement therapy	1229 (16.9)	56 (2.5)	107 (10.1)	46 (6.3)	153 (13.1)	866 (43.0)
Capillary leak	634 (8.7)	9 (0.4)	29 (2.8)	6 (0.8)	36 (3.1)	554 (27.5)
Catecholamine requirement	2887 (39.6)	244 (11.1)	388 (36.7)	138 (18.9)	230 (19.7)	1886 (93.7)
Microcirculatory dysfunction (8)	1435/5646 (97.1)	55 (2.5)	106 (10.0)	51 (7.0)	190 (16.3)	1033 (51.3)
Clinical suspicion	675 (9.3)	12 (0.5)	30 (2.8)	11 (1.5)	82 (7.0)	540 (26.8)
Recapillarization time > 2 s	122 (1.7)	6 (0.3)	5 (0.5)	–	2 (0.2)	109 (5.4)
Hyperlactatemia (> 2 mmol/L)	1009 (13.8)	46 (2.1)	76 (7.2)	35 (4.8)	110 (9.4)	742 (36.9)
(ScvO_2_) > 80%	395 (5.4)	19 (0.9)	25 (2.4)	6 (0.8)	55 (4.7)	290 (14.4)
Preceding 24-h trend (2)						
Improved	1440 (19.8)	491 (22.3)	241 (22.8)	170 (23.2)	248 (21.3)	290 (14.4)
Deteriorated	1,062 (14.6)	137 (6.21)	158 (15.0)	86 (11.7)	131 (11.2)	547 (27.2)
Unchanged	4665 (64.0)	1574 (71.4)	655 (62.0)	474 (64.8)	788 (67.5)	1174 (58.3)
Expected 24-h trend (10)						
Improve	1050 (14.4)	376 (17.0)	161 (15.2)	130 (17.8)	153 (13.1)	230 (11.4)
Deteriorate	493 (6.7)	60 (2.7)	74 (7.0)	30 (4.1)	38 (3.3)	291 (14.5)
No change	5542 (76.0)	1754 (79.5)	807 (76.4)	565 (77.2)	957 (82.0)	1459 (72.5)
Among (1)						
3 most severely ill ICU patients	1030 (14.1)	95 (4.3)	112 (10.6)	21 (2.9)	128 (11.0)	670 (33.3)
3 least severely ill ICU patients	1029 (14.1)	649 (29.4)	132 (12.5)	125 (17.1)	58 (5.0)	58 (2.9)
Microbiology testing						
Blood cultures	1713 (23.5)	404 (18.3)	285 (27.0)	152 (20.8)	246 (21.1)	607 (30.2)
Bronchial lavage	556 (7.63)	65 (2.95)	74 (7.01)	44 (6.01)	104 (8.91)	262 (13.0)
Antimicrobial therapy	3775 (51.8)	274 (12.4)	238 (22.5)	519 (70.9)	894 (76.6)	1789 (88.9)

*ScvO*_*2*_ central venous oxygen saturation

^a^Absolute numbers for explicitly affirmative and non-affirmative responses to GTSQ items are separated by a slash, and the percentage of the column category (n) for their sum is given in parentheses. Else, numbers for affirmative replies or counts (% of column category) are given

^b^The working diagnosis (Item 3) was not assigned in 118 (1.62%) of all 7291 edited questionnaires

^c^“Endocarditis” was included into Item 5 only from 01/08/2016 onwards in altogether 7025 GTSQs

^d^Not chronic preexisting

Proportions of blood cultures and bronchial lavages were 1.6- and 2.8-times higher, respectively, and antimicrobial orders were 5.2-times higher in association with any sepsis label than with a non-sepsis label (Table 3) (p < 0.0001 for all comparisons). Additional file 1: Fig. S1 summarizes the distributions of average values of 12 selected clinical parameters in the rating intervals for all 7291 edited GTSQs across the five working diagnosis categories. Sepsis labels, especially *septic shock*, overall correlated with worse clinical parameters.

#### Labels and proxies for suspicion of infection

Suspicion of infection (Item 4) was documented in 70 GTSQs with a *neither SIRS nor sepsis* label (3.2%) and in 87 with a *SIRS* label (8.2%) (Table 3), which were from 52 and 69 encounters, respectively. Almost all of the 157 GTSQs with a suspicion of infection and concomitant non-sepsis label were associated with a SOFA score ≥ 2 (153 GTSQs from 108 encounters). Although clearly associated with sepsis labels, antimicrobial therapy and blood culture orders were also documented in 12.4% and 18.3% of GTSQs, respectively, with a *neither SIRS nor sepsis* label and in 22.5% and 27% with a *SIRS* label.

#### Focus localization

A focus of infection (Item 5) was marked as present in 10.6% of all edited GTSQs with a *neither SIRS nor sepsis* label, 22.0% with a *SIRS* label, and 95.1% with any sepsis label (Table 3). These fractions were contributed by 89, 92, and 347 encounters, respectively. The relative frequencies of both confirmed and suspected focus localizations among all edited GTSQs increased from *SIRS* to *septic shock* independent of localization.

The thorax was marked as a suspected or confirmed focus in 6.4% of all GTSQs with a non-sepsis label, in over one third of all those with a *sepsis* label, and both in over half of those with a *severe sepsis* and *septic shock* label followed by the abdomen with somewhat lower proportions, respectively (Table 3). The selection rate for each of the remaining focus localizations among all GTSQs was < 1.6% for the combined non-sepsis labels and < 10% for the combined sepsis labels.

Notably, 4.9% of all GTSQs with any sepsis label, corresponding to 67 encounters, were at the same time answered in the negative for focus of infection. Likely, the infectious source was considered already under control. In 74.5% of all non-sepsis labels with concomitant suspicion of infection (Item 4) (88 encounters) the presence of a focus was also negated.

#### Source control

The relative frequency of performance of infectious source control (Item 6) was ten times higher in conjunction with a sepsis label (10.2%) than with a non-sepsis label (1.0%) (Table 3), corresponding to 154 and 30 encounters, respectively. The control measure was mostly surgical, especially in combination with a *septic shock* label, where its frequency was 5.8-fold higher than all other measures together. In 3.7% of source control positive GTSQs, contributed by 16 encounters, the answer to the question for a focus of infection (Item 5) was *No* suggesting that the measure was considered already successful.

#### Acute organ dysfunction

**Fig. 6:**
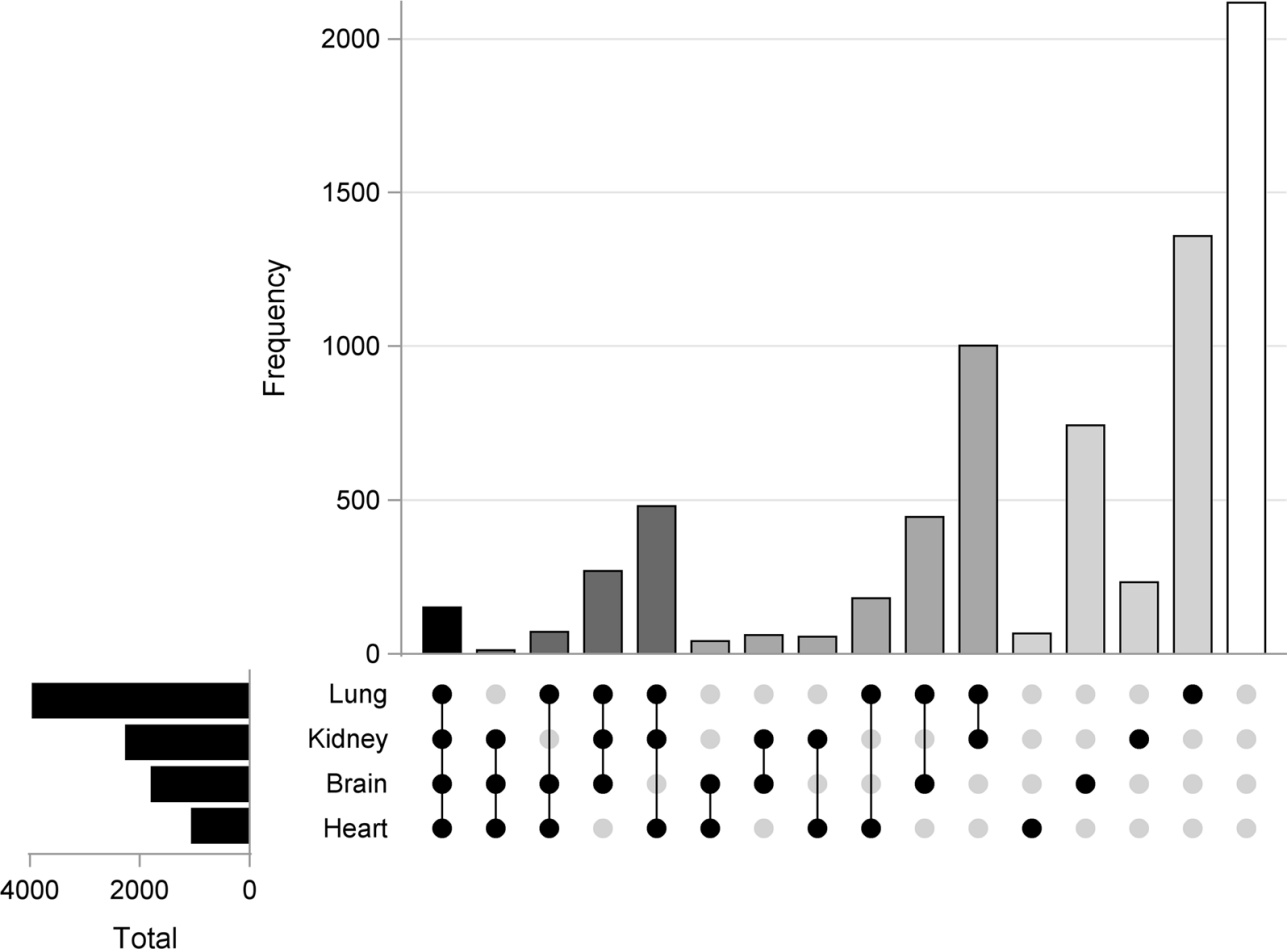
Upset plot of the four most frequent acute organ dysfunction labels at the GTSQ level (Item 9). Frequencies for all possible combinations of organ dysfunction labels are displayed as gray shaded bars: no label (white), one label (light gray), two labels (gray), three labels (dark gray) and four labels (black). Horizontal bars represent cumulative numbers

Multiple organ dysfunction is a common cause of death in sepsis. Therefore, we included the labelling of organ dysfunction as a central item (Item 9). Acute organ dysfunction was indicated in around half of all 7291 edited GTSQs for the lung, one third for the kidney, a quarter for the brain, and in about one out of seven for the heart (Table 3 and Fig. 6). Lung and kidney formed the most frequent combination. In 22.6% of all edited GTSQs, the presence of acute organ dysfunction was negated.

**Fig. 7:**
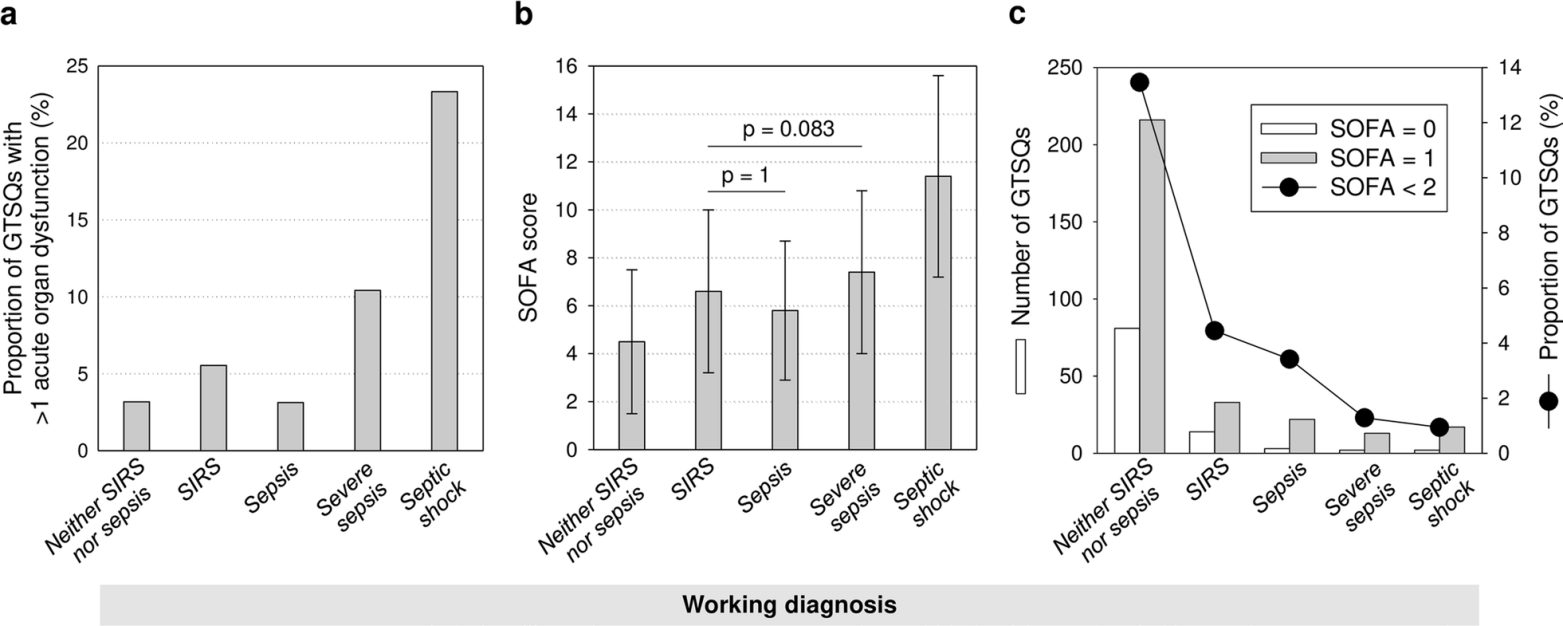
Degrees of organ dysfunction across all edited GTSQs by working diagnosis (Item 3). **a** All between-working diagnosis differences for the proportions of all GTSQs with more than one acute organ dysfunction (Item 9) were statistically significant (p < 0.001 from Chi-squared test). **b** The same applied to the SOFA scores (p < 0.005 from a generalized linear mixed model) except, as indicated by the p-values, for the *SIRS* versus *sepsis* and *SIRS* versus *sever sepsis* comparisons. SOFA scores are displayed as mean values (bars) with standard deviations (whiskers). **c** Absolute numbers of GTSQs with a concurrent SOFA score of 0 (white bars) and 1 (gray bars) and their combined proportions (black dots) are plotted together for each working diagnosis. The proportions in **c** are connected by lines to aid visual comparison between working diagnoses

The number of acute organ dysfunctions exceeded one in 45.6% of all edited GTSQs. The presence of any organ dysfunction label (acute and/or chronic) was negated in only 1.8% of all GTSQs with a *severe sepsis* and *septic shock* label (Item 3) together. Proportions of GTSQs with more than one acute organ dysfunction and mean SOFA scores showed similar profiles across working diagnosis categories (Fig. 7a, b). Differences in SOFA between *SIRS* and *sepsis* as well as *SIRS* and *severe sepsis* did not reach statistical significance (Fig. 7b). SOFA scores below 2 were overall infrequent (Fig. 7c). Values of 0 and 1, respectively, were scored for only 2.9% and 7.6% of all GTSQs in the non-sepsis and only 0.2% and 1.3% in the sepsis categories. A SOFA of 2 or higher was associated with 89.5% of all non-sepsis and 98.5% and all sepsis GTSQs.

**Fig. 8:**
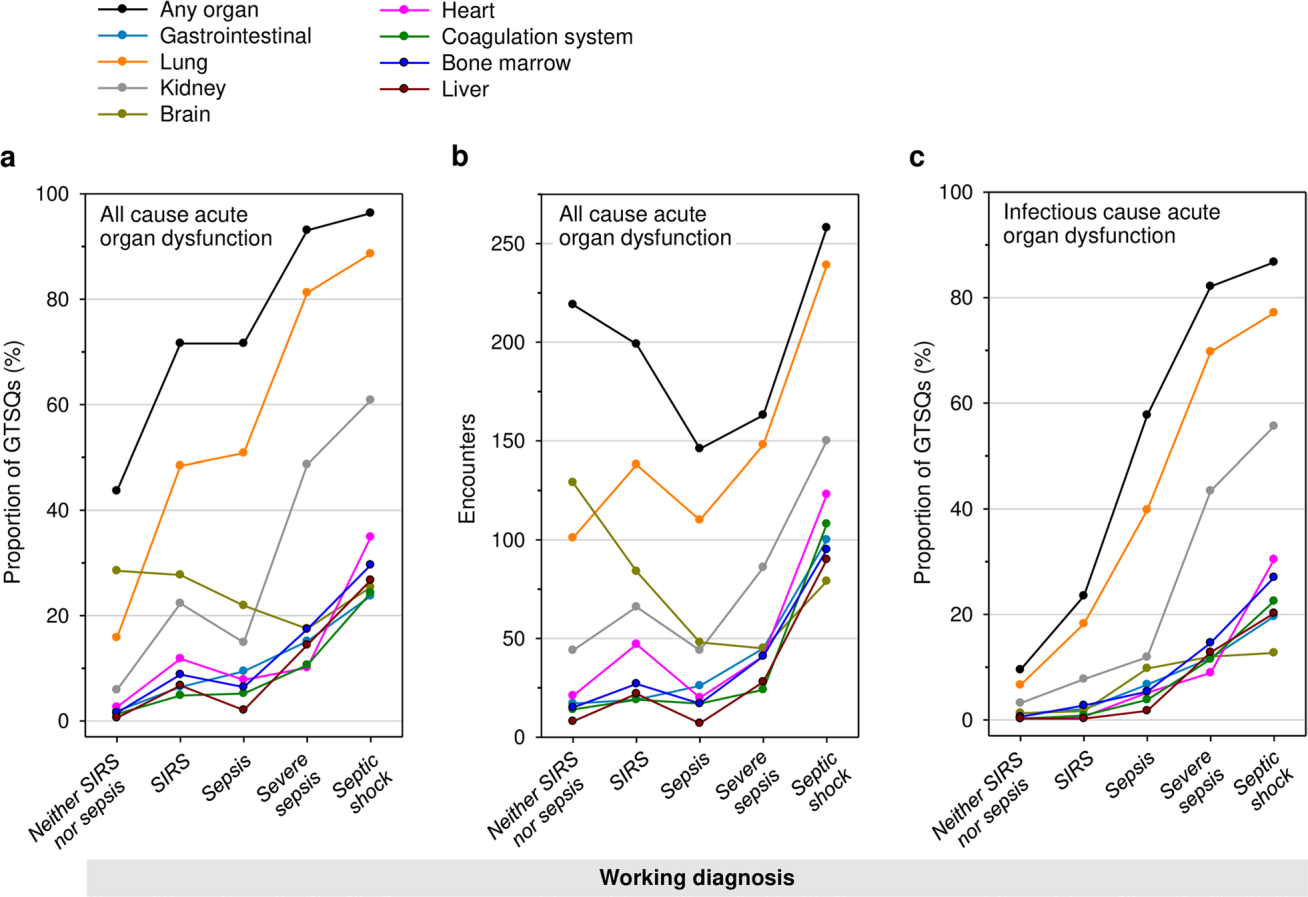
Individual acute organs dysfunction labels (Item 9) by working diagnosis (Item 3). Proportions of all GTSQs with any (black line-symbol) and with specific (colored line-symbol) acute organ dysfunctions for **a** all causes and **c** infectious causes by working diagnosis. **b** The numbers of encounters underlying the proportions for all cause organ dysfunctions in **a**. Data points are connected by lines to aid visual comparison between working diagnoses

Proportions of all edited GTSQs with an acute lung dysfunction label steadily increased with presumptive working diagnosis severity, while those of GTSQs with other acute organ dysfunction labels, except for brain, increased consistently only from *sepsis* on (Fig. 8a). For each acute dysfunction but lung and gastrointestinal, proportions were higher for *SIRS* than for *sepsis*. The numbers for the encounters (not restricted to complete encounters) contributing the respective GTSQs to these proportions were overall distributed similarly with one exception (Fig. 8b). The number of 219 encounters contributing any acute organ dysfunction label in conjunction with a *neither SIRS nor sepsis* label was relatively high, driven by encounters with a label for acute brain dysfunction.

The proportions of individual acute organ dysfunctions judged to have an infectious cause from all GTSQs were below 12% for each individual organ but the lung (42.3%) and the kidney (26.5%). Individual organ dysfunctions were rated as of non-infectious cause in less than 7% of all GTSQs each except for brain (23.0%) and lung (13.0%) (Additional file 1: Table S4). Proportions of infectious cause organ dysfunction across the individual working diagnoses increased throughout with presumptive severity of illness for all organ systems (Fig. 8c). Among the GTSQs with any infectious cause acute organ dysfunction and a non-sepsis working diagnosis label, 244 were from complete encounters, and 54 of these were from altogether 14 sepsis-free encounters (according to encounter level rating).

Overall, associations of SOFA scores and organ dysfunction labels with working diagnosis labels does not support a generally higher illness severity in sepsis than in SIRS.

#### Concurrent acute organ dysfunction and focus localization

**Fig. 9:**
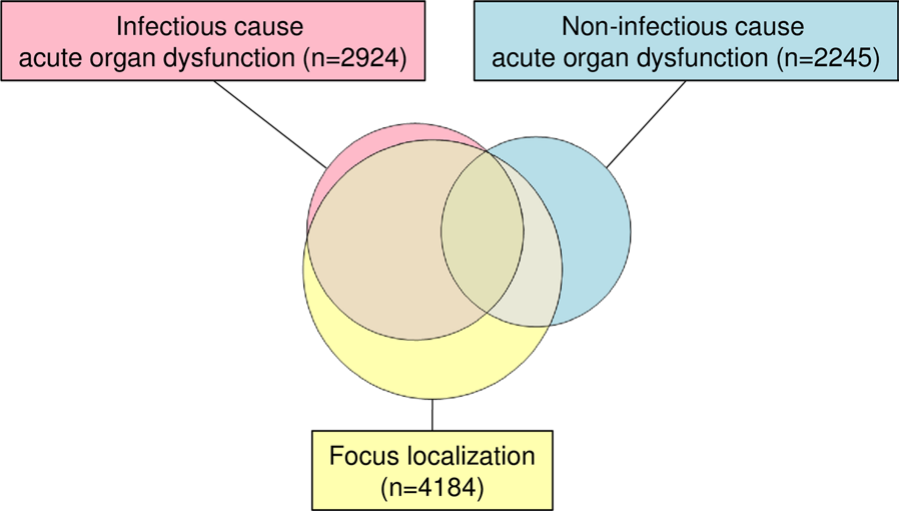
Concurrent labelling of focus localization and acute organ dysfunction. Venn diagram showing the relationships between the groups of GTSQs with a positive label for acute organ dysfunction of infectious (rose) and non-infectious (blue) cause (Item 9) and for a positive focus localization label (yellow) (Item 5)

Infection bears the risk of organ dysfunction and vice versa. Among all 5267 GTSQs with any acute organ dysfunction label (Item 9, Table 3), at least one dysfunction was judged of infectious cause in 55.5% and at least one of non-infectious cause in 42.6%. Because dysfunction was classified as of infectious or non-infectious cause for every organ system individually, a given GTSQ could be counted in both of these subgroups. In 743 GTSQs, acute dysfunction of at least one organ was classified as infectious and that of at least one as non-infectious. Figure 9 represents these relationships as Venn diagram. Of the GTSQs with infectious and non-infectious cause acute organ dysfunction labels, respectively, 93.4% and 44.1% were associated with a positive focus of infection (Item 5). Considering complete encounters only, encounters with at least one sepsis working diagnosis (Item 3) contributed over 90% of the GTSQs in each region of the Venn diagram as shown in Fig. 9 with one exception. Namely, 43.7% of the GTSQs with exclusively non-infectious cause acute organ dysfunction and no positive focus localization label were from sepsis-free encounters.

**Fig. 10:**
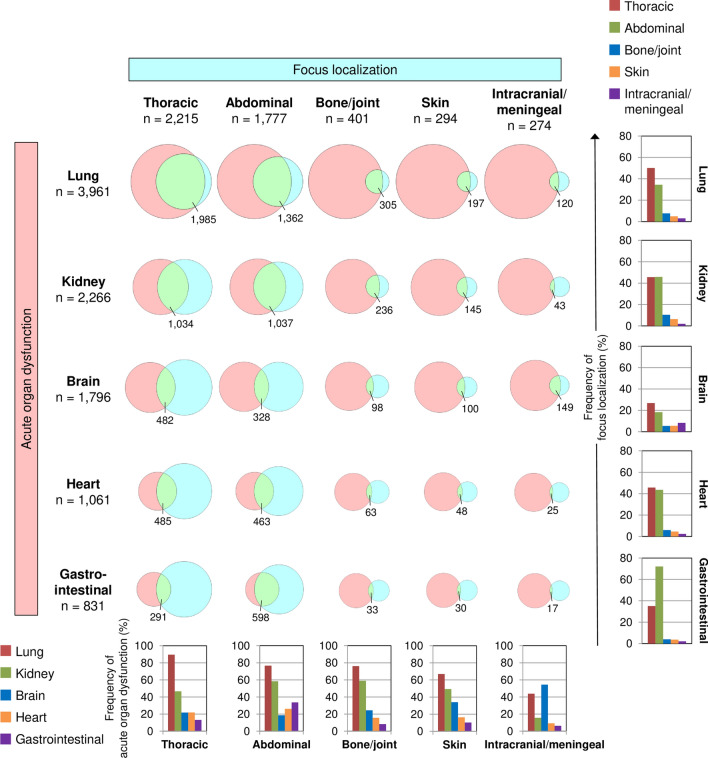
Co-occurrence of acute organ dysfunction (Item 9) and focus localization (Item 5) labels. The frequencies of all GTSQs with a combination of a label for lung, kidney, brain, heart or gastrointestinal dysfunction (rose spheres) and a thoracic, abdominal, bone/joint, skin, or intracranial/meningeal focus localization (cyan spheres) are indicated in the respective Venn diagrams. The bar charts at the right summarize the overlaps of the combinations as proportions of dysfunction labels by organ and at the bottom as proportions of focus localization labels by localization. Because varying numbers of organs could be labelled as dysfunctional and various numbers of localizations be identified as foci, the sum of the bars shown may exceed 100%. And because only a selection of dysfunctions and localizations is shown, the sum may not reach 100%

The data on all possible pairs of acute organ dysfunction and focus localization labels from all edited GTSQs is tabularized in Additional file 1: Table S5. Figure 10 summarizes the relations for the four most prevalent acute organ dysfunctions (lung, kidney, brain and heart) and focus localizations (thoracic, abdominal, bone/joint and skin). Gastrointestinal dysfunction and intracranial/meningeal focus localization were added to this selection because they correspond to abdominal focus and brain dysfunction, respectively. The absolute overlap was largest for lung/thoracic. In agreement with overall organ dysfunction frequencies, lung dysfunction was proportionally most frequent in each of the focus localizations followed by kidney dysfunction except for intracranial/meningeal focus, which was proportionally most frequently associated with brain followed by lung dysfunction. Also, the highest and second highest overall prevalence of thoracic and abdominal foci, respectively, agreed with their dominance across all organ dysfunctions. Proportionate frequencies for thoracic and abdominal focus localizations were highest and second highest, respectively, in lung and brain dysfunction and were close to equal in kidney and heart dysfunction. Gastrointestinal dysfunction, however, was associated more than twice as often with an abdominal focus (in 72%) than a thoracic focus (in 35%).

#### Circulatory problems

Multiple organ dysfunction in sepsis is commonly associated with tissue ischemia. Therefore, we included labelling of circulatory problems in the GTSQ. Macrocirculatory abnormalities (Item 7) were indicated in 42.0% of all edited GTSQs (Table 3). Their proportions were 11.6% for GTSQs with a *neither SIRS nor* sepsis label followed by 22.1% with a *sepsis* label*,* 28.4% with a *severe sepsis* label, 38.1% with a *SIRS* label*,* and 94.8% with a *septic shock* label (Item 3) (p < 0.0001 for *septic shock* versus all other labels and for *SIRS* versus *sepsis*). These percentages corresponded to 97, 139, 70, 100, and 262 contributing encounters, respectively. Macrocirculatory abnormalities were indicated at least once in 24.5% and 84.9% of the complete encounters in the combined non-sepsis and sepsis categories, respectively. Of all 2012 *septic shock* labels, 3.3% coincided with an explicit label for absence of macrocirculatory abnormality.

**Fig. 11:**
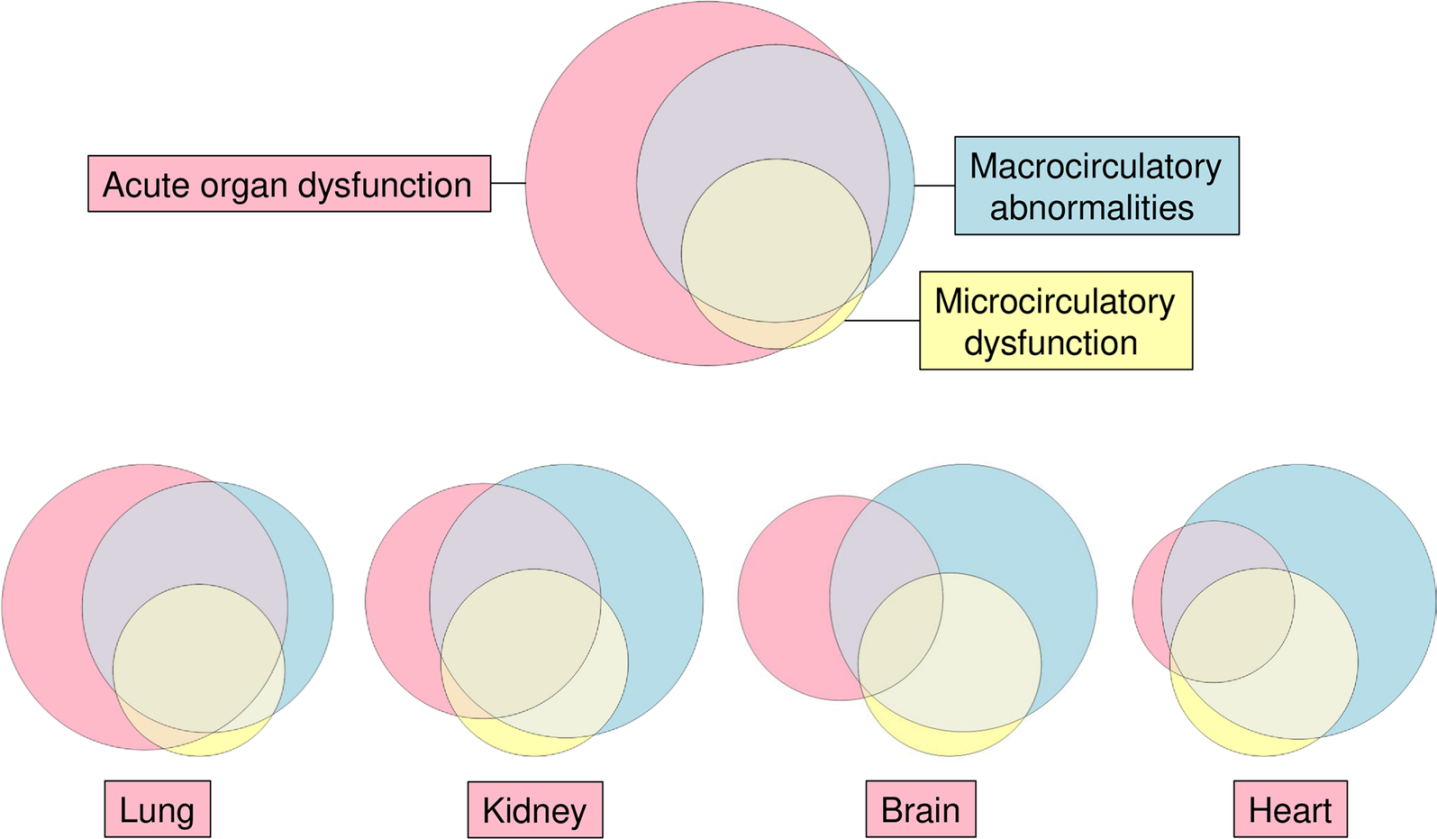
Co-occurrence of circulatory problems and acute organ dysfunction. Venn diagrams of GTSQ labels for acute organ dysfunction (Item 9), macrocirculatory abnormalities (Item 7), and microcirculatory dysfunction (Item 8). Acute dysfunction of any organ is shown on top and, separately, for the four most prevalent acute organ dysfunctions below

Microcirculatory dysfunction (Item 8, Table 3) was judged present in 19.7% of all GTSQs and 33.3% of the complete encounters, and in half of the GTSQs and 76.5% of the complete encounters with a *septic shock* label. Of all 1435 GTSQs with a microcirculatory dysfunction label, 11.9% had no concurrent macrocirculatory abnormality label (Fig. 11).

Acute organ dysfunction labels (Item 9) coincided with macrocirculatory abnormalities in 53.8% and with microcirculatory dysfunction in 26.3% (Fig. 11). Viewed by organ, frequencies of macro- and microcirculatory complications (respectively) increased as expected from brain (48.1% and 20.2%), to lung (61.6% and 31.4%), kidney (68.3% and 41.8%), and heart dysfunction (84.1% and 51.9%).

Taken together, while the strong association of the label for macrocirculatory abnormalities with *septic shock* was highly predictable, its higher proportion in *SIRS* than in *sepsis* and especially *severe sepsis* was unexpected.

#### Course of disease

Consecutive working diagnosis labels to identify day-to-day transitions were available 5809 times. Labels remained unchanged in 82.7% of these transitions. The change from *septic shock* to *severe sepsis* was most frequent (3.1%) followed by *severe sepsis* to *septic shock* (1.8%) and *neither SIRS nor sepsis* to *SIRS* and vice versa (1.5% each).

The overall clinical picture during the preceding 24 h was rated as changed in 34.4% of all GTSQs (Item 2, Table 3). For each working diagnosis label (Item 3) excluding *septic shock*, it was judged as improved in close to 22% and as deteriorated in 6–15%. The proportions were reverse for the *septic shock* label with 14.4% improved and 27.2% deteriorated. Proportions of label transitions from *neither SIRS nor sepsis* to any form of sepsis with a concurrent improvement in the overall clinical picture or in the opposite direction with concurrent deterioration were below 0.6% each. Also, as expected, proportions of label transitions within the sepsis spectrum to a more and to a less severe form in association with improvement and deterioration, respectively, were both below 0.5%.

### Daily ranking of illness severity

Considering the proportion of GTSQs with a given working diagnosis, *septic shock* was the label that was, in relative terms, most frequently associated with an assignment to the three most severely ill patients (33.3%) and least frequently with the three least severely ill (2.9%) (Item 1, Table 3). As also expected, *neither SIRS nor sepsis* was the label that coincided most frequently with an assignment to the three least severely ill (29.4%). *Sepsis* was least frequently associated with assignment to the three most severely ill (2.9%) followed by *neither SIRS nor sepsis* (4.3%). Notably, in almost equal proportions of the GTSQs with a *SIRS* (10.6%) and with a *severe sepsis* (11.0%) label a patient was assigned to the three most severely ill (no statistical difference). The 3.7-fold higher proportion of GTSQs with this assignment and a concurrent *SIRS* label compared to *sepsis* (p < 0.0001) and the similar proportion compared to septic shock were unexpected.

### Subgroup analysis of neurosurgical referrals

Immune and central nervous system (CNS) functions interact in a reciprocal fashion [53], and pre-existing CNS damage has the potential to influence outcomes. Because neurosurgical referrals accounted for almost half of our encounters (Table 2), we examined whether the relatively high illness severity associated with the *SIRS* label compared to *sepsis* and *severe sepsis* was a particular characteristic of this referral group in a subgroup analysis. We did not consider specific neurosurgical diagnoses. Five complete encounters without referring department were excluded.

#### Encounter level

**Fig. 12:**
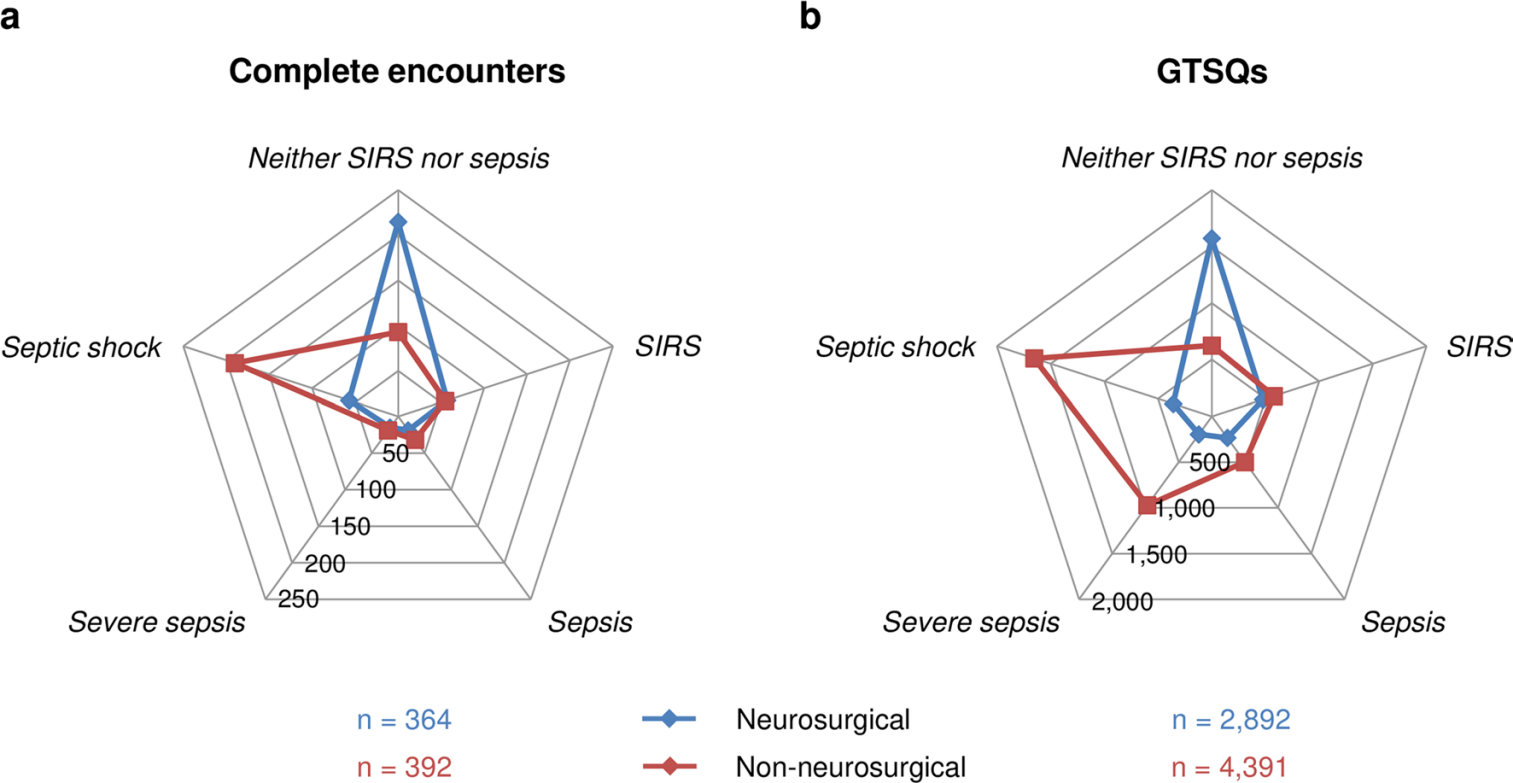
Working diagnosis distributions in a subgroup analysis. Radar chart of working diagnosis (Item 3) distributions for **a** 364 neurosurgical (red) and 392 non-neurosurgical (blue) referrals (encounter level) and **b** 2892 neurosurgical and 4391 non-neurosurgical GTSQs (GTSQ level)

Additional file 1: Table S6 summarizes the characteristics of complete encounters for neurosurgical and non-neurosurgical referrals in analogy to Table 2. Age and age group distributions were highly similar but the proportion of women was higher in the neurosurgery (46.4%) than in the non-neurosurgery group (32.9%) (p = 0.0001). Figure 12a displays the distributions of the different working diagnosis categories (Item 3) for complete encounters from both subgroups. The majority (59.1%) of neurosurgical referrals were assigned to the *neither SIRS nor sepsis* category and 25.0% to any sepsis category. Conversely, the majority (61.5%) of non-neurosurgical referrals had at least one sepsis label, mainly septic shock, and 23.7% remained at the *neither SIRS nor sepsis* category level. In either subgroup, the fraction of encounters in the *SIRS* category was comparable (15.7% for neurosurgical and 14.0% for non-neurosurgical). Neurosurgical referrals accounted for 27 of our 205 present-on-admission sepsis (13.2%) and for 56 of our 109 incident sepsis cases (51.4%).

Overall ICU mortality was lower in the neurosurgery than the non-neurosurgery group (16.5 vs. 21.9%, Additional file 1: Table S6). ICU mortalities were low in both groups for the *neither SIRS nor sepsis* (7.4% and 4.3%, respectively) and the *severe sepsis* (6.7% and 5.3%) categories and highest each for *septic shock* (45.6% and 37.9%). Interestingly, all 19 neurosurgical referrals in the *sepsis* category survived and 17 out of 57 (29.8%) in the *SIRS* category of this referral group did not, whereas the *sepsis* and *SIRS* encounters in the non-neurosurgery group had very similar ICU mortalities (9.1% and 9.4%, respectively). For the neurosurgery group, ICU mortality for *SIRS* showed no statically significant difference compared to all sepsis categories together while it was lower for the non-neurosurgery group (p < 0.001).

**Fig. 13:**
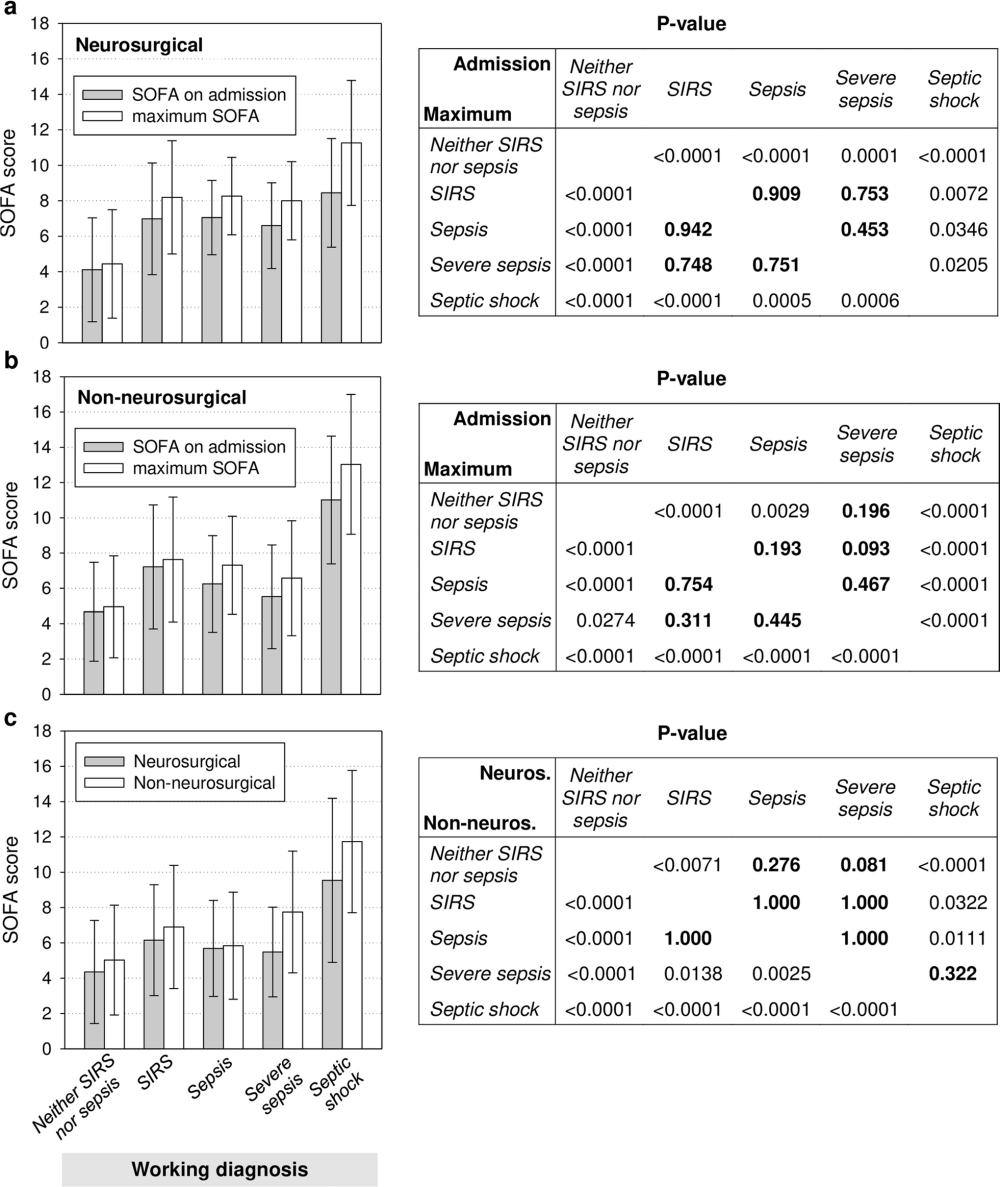
SOFA scores for neurosurgical and non-neurosurgical referrals by working diagnosis (Item 3). Mean values for SOFA scores (bars) with standard deviations (whiskers) on admission (gray) and for maximum SOFA scores (white) from complete encounters are shown for **a** neurosurgical and **b** non-neurosurgical referrals. The tables to the right show the corresponding p-values from the Mann–Whitney-U test for all between-working diagnosis differences in on-admission and maximum SOFA scores, respectively, above and below the diagonal. **c** Mean SOFA scores (bars) ± standard deviations (whiskers) of all edited GTSQs are displayed for neurosurgical (gray) and non-neurosurgical (white) referrals. The table shows p-values from a generalized linear mixed model for all between-working diagnosis differences in neurosurgical and non-neurosurgical referrals, respectively, above and below the diagonal. In the tables (**a**–**c**), the absence of statistically significance is highlighted by p-values printed in bold

CCI values in the *neither SIRS nor sepsis* and the *SIRS* categories, separately and together, were lower than in the combined sepsis categories for neurosurgical (all p-values < 0.01) but not for non-neurosurgical referrals (Additional file 1: Table S6). In both referral groups, admission and maximum SOFA scores showed no statistically significant differences between *SIRS*, *sepsis*, and *severe sepsis* (Fig. 13a, b).

### GTSQ level

Neurosurgical referrals accounted for 39.7% of all 7291 edited GTSQs. Additional file 1: Table S7 summarizes responses to the GTSQ Items and according SOFA scores by working diagnosis (Item 3) for both referral groups separately in analogy to Table 3. Neurosurgical referrals contributed altogether 2892 and non-neurosurgical 4391 GTSQs of which 27.3% and 71.1%, respectively, carried a sepsis label. The working diagnosis distribution profile for neurosurgical GTSQs (Fig. 12b) closely matched the corresponding profile for complete encounters (Fig. 12a). For non-neurosurgical referrals, by contrast, the relative frequency of a *severe sepsis* label was higher on the GTSQ level (Fig. 12b) than that of the *severe sepsis* category on the encounter level (Fig. 12a). In non-neurosurgical GTSQs, SOFA scores differed between all working diagnoses except for *SIRS* and *sepsis*, whereas there were fewer between-working diagnosis differences in neurosurgical GTSQs (Fig. 13c).

**Fig. 14:**
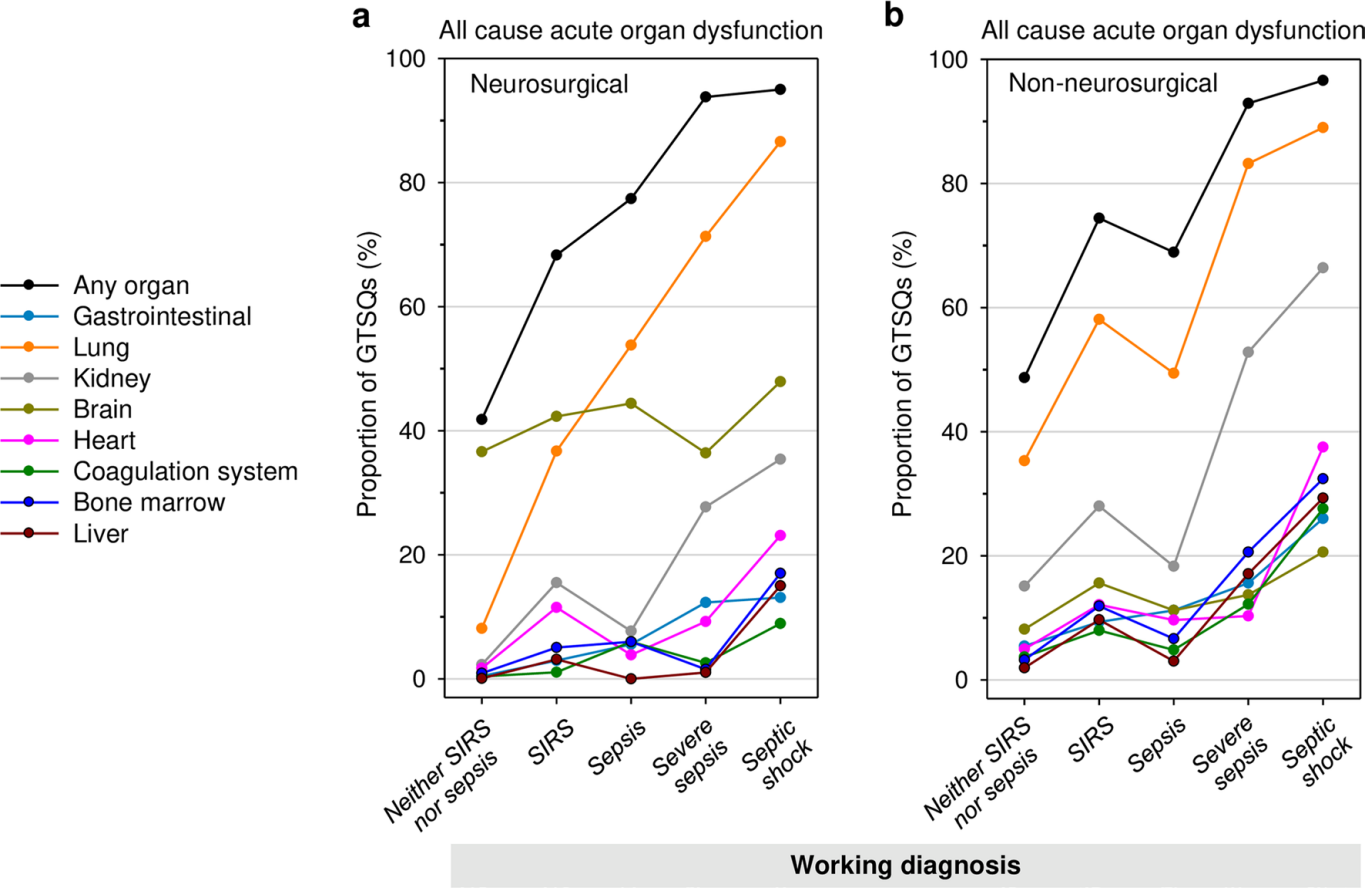
Individual acute organ dysfunction labels by working diagnosis (Item 3) in the subgroup analysis. The proportions of all GTSQs with any (black line-symbol) and with specific (colored line-symbol) organ dysfunctions are plotted by working diagnosis for all causes in **a** neurosurgical and **b** in non-neurosurgical referrals. Data points are connected by lines to aid visual comparison between working diagnoses

Figure 14 shows the profiles for the proportions of all cause acute organ dysfunction labels (Item 9) across working diagnoses for all edited GTSQs in the neurosurgical and the non-neurosurgical referral groups. Profiles were overall similar to the ones for all referrals together (Fig. 6a) except for brain dysfunction, which was relatively frequent across all working diagnosis labels in the neurosurgery (36.4–47.9%) compared to the non-neurosurgery group (8.2–20.6%).

The editing rates for both macro- and microcirculatory complications (Items 7 and 8) were 97% for both the neuro- and non-neurosurgical GTSQs (Additional file 1: Table S7). In the neurosurgical GTSQs, macrocirculatory abnormalities were, however, proportionally negated 2.4-times more often than they were affirmed compared to slightly more affirmative than negative replies in the non-neurosurgical GTSQs. Accordingly, microcirculatory dysfunction was negated even 11.5-times more often than affirmed in the neurosurgical GTSQs compared to 2.5-times in the non-neurosurgical GTSQs. The profiles for the proportions of macro- and microcirculatory abnormalities across the different working diagnoses in the GTSQs from both referral groups resembled the global profile for all 7291 edited GTSQs (Table 3). In both subgroups, we especially note higher proportions of macrocirculatory abnormalities in *SIRS* than in *sepsis* (1.4-fold for neurosurgical, p = 0.003, and 1.9-fold for non-neurosurgical, p < 0.0001). The proportion in *SIRS* was also higher than in *severe sepsis* for non-neurosurgical GTSQs (1.5-fold, p < 0.0001) while the two showed no statistically significant difference in neurosurgical GTSQs (p = 0.2).

#### Daily ranking of illness severity

Results from the ranking of illness severity (Item 1) for neuro- and non-neurosurgical GTSQs (Additional file 1: Table S7) resembled the global profile (Table 3). In the neurosurgical group, the proportion of GTSQs associated with assignment to the three most severely ill patients was almost 4-times higher for *SIRS* (13.6%) than both *sepsis* (3.4%) (p < 0.0001) and *severe sepsis* (3.6%) (p = 0.0001). For the non-neurosurgical GTSQs, the corresponding proportions were 3.1-times higher for *SIRS* (8.1%) than *sepsis* (2.6%) (p < 0.0001) but 1.5-fold lower for *SIRS* than *severe sepsis* (12.4%) (p < 0.0001).

Table 4 summarizes the principal differences between neurosurgical and non-neurosurgical referrals from the subgroup analysis.

**Table 4:** Summary of principle subgroup differences and similarities

Feature	Subgroup differences and similarities
Mortality	The *SIRS* compared to the *sepsis* working diagnosis category was associated with higher mortality in neurosurgical encounters, while mortality in both categories was similar in non-neurosurgical encounters (Additional file 1: Table S6)
SOFA score	SOFA scores in both referral groups showed overall no differences between *SIRS and sepsis* as well as *severe sepsis* except for higher values with non-neurosurgical GTSQs carrying a *severe sepsis* label (Fig. 13)
Acute organ dysfunction (Item 9)	The proportion of GTSQs with a label for acute organ dysfunction was higher in neurosurgical GTSQs with a *SIRS* than *sepsis* label, mainly due to lung and brain dysfunction (Fig. 14a). In the non-neurosurgery GTSQs by contrast, acute organ dysfunction declined from *SIRS* to *sepsis* for almost all organ systems (Fig. 14b)
Macrocirculatory abnormalities (Item 7)	Circulatory problems were much more prevalent among the non-neurosurgical GTSQs (Additional file 1: Table S7). Nevertheless, in the GTSQs from both referral groups macrocirculatory abnormalities were more frequently associated with *SIRS* than *sepsis*, while a more frequent association of macrocirculatory abnormalities with *SIRS* than with *severe sepsis* was only seen in the non-neurosurgical GTSQs
Daily ranking of illness severity (Item 1)	In both referral groups, GTSQs with a *SIRS* label compared to a *sepsis* label were relatively more often associated with assignment to the three most severely ill patients (Additional file 1: Table S7)

### Validation of Sepsis-1/2 and Sepsis-3 clinical criteria against ground truth labels

We applied clinical criteria for both sepsis consensus definitions to the EHRs of our complete encounters. We included all 109 encounters with incident sepsis and 205 with on-admission sepsis and excluded the 18 sepsis encounters that were not assigned to either of these two categories. The four encounters without any working diagnosis label as well as one sepsis-free encounter with missing working diagnosis labels on 4 consecutive days were excluded. Among the remaining 424 included sepsis-free encounters, only three were missing a working diagnosis label on 2 consecutive days and 12 on a single day.

In a first step, a suspicion of infection was identified at least once in altogether 281 encounters, of which 262 also had a working diagnosis label for sepsis (Item 3). In a second step, the timely presence of SIRS and SOFA ≥ 2 was derived to determine the presence of sepsis according to Sepsis-1/2 and Sepsis-3, respectively. The relative frequency of Sepsis-1/2 was 32.9% and of Sepsis-3 33.6%, representing essentially the same set of encounters (K_α_ = 0.957). In our GTSQ survey by comparison, 42.6% of the included complete encounters were assigned any sepsis label and 36.0% a *sever sepsis* or *septic shock* label. In the following comparisons, ground truth labels represent the reference class.

Considered as tests for the detection of sepsis as a dichotomous characteristic of an encounter, Sepsis-1/2 and Sepsis-3 clinical criteria showed substantial agreement with ground truth labels (K_α_ between 0.63 and 0.65, Additional file 1: Appendix S2). SIRS predicted any of the three sepsis labels with a sensitivity of 68.8% and specificity of 93.6% and SOFA ≥ 2 with 67.2% and 93.6%, respectively. A label for *severe sepsis* or *septic shock* was predicted by SIRS with 72.9% sensitivity and 89.6% specificity and by SOFA ≥ 2 with 72.6% and 90.5%, respectively.

**Fig. 15:**
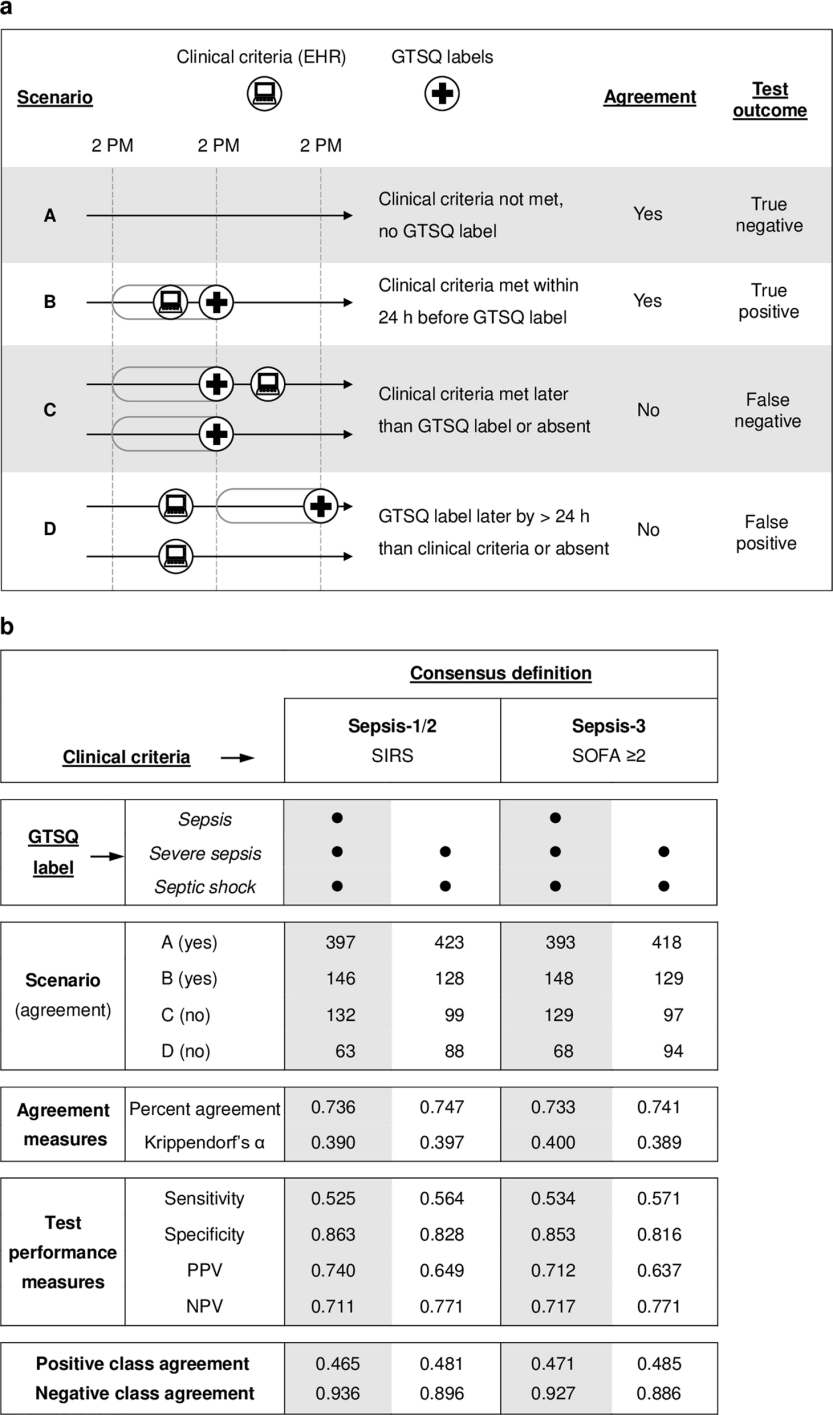
Comparison of clinical criteria to ground truth labels for detection of the first sepsis episode. Sepsis onset in our 761 complete encounters was determined according to clinical criteria (computer icon) for Sepsis-1/2 (SIRS) and Sepsis-3 (SOFA ≥ 2). This point was compared to the first occurrence of a GTSQ label for sepsis (cross icon) considering either all three sepsis categories together or only *severe sepsis* and *septic shock*. **a** Identifies the four scenarios and according ratings, based on which agreement and test performance were evaluated. **b** Enumerates the results of the comparisons and the statistical measures of agreement and test performance. The 95% confidence intervals for all test performance measures were within ± 7%. *PPV* positive predictive value, *NPV* negative predictive value

The clinical diagnosis of sepsis is however time-critical. Therefore, we also assessed the temporal agreement between sepsis onset detected by clinical criteria and by ground truth labels (Fig. 15a). Agreement was given if clinical criteria were neither met nor a ground truth label for sepsis existed (scenario A), or if clinical criteria detected sepsis onset within the 24 h prior to the first sepsis label (scenario B). Notably, it is fair to expect clinical criteria to precede expert labels (scenario B), because the rules for identifying a suspicion of infection and for the determination of SIRS and SOFA both consider EHR entries that lie ahead of the eventual sepsis onset. Clinical criteria were considered overdue if they lagged behind the first sepsis label, independent of the time elapsed, or were absent (scenario C). Clinical criteria were regarded untimely if they preceded the first sepsis label by more than 24 h or were met in the absence of a sepsis label (scenario D). Overdue and untimely detection was counted as no agreement.

Clinical criteria for Sepsis-1/2 as well as Sepsis-3 yielded only fair agreement with ground truth labels for sepsis onset, regardless of whether *sepsis*, as the least severe working diagnosis category in the sepsis spectrum, was included or not in the comparison (Fig. 15b). Requiring a change in SOFA by at least two points instead of SOFA ≥ 2 did not change the agreement for Sepsis-3. At least 92% of the encounters identified each through the clinical criteria for Sepsis-1/2 and Sepsis-3 in a given scenario (A–D) were identical indicating very high concordance between the two consensus definitions. Accordingly, Sepsis-1/2 and Sepsis-3 clinical criteria all performed very similarly as diagnostic tests with sensitivities between 53 and 57% and specificities between 82 and 86%, and positive predictive values (PPVs) between 64 and 74% and negative predictive values (NPVs) between 72 and 77%. Both consensus definitions were, however, more likely to correctly exclude (NPV) than include (PPV) the presence of *severe sepsis* or *septic shock* by 12.2% (Sepsis-1/2) and 13.4% (Sepsis-3) compared to little differences for the full sepsis spectrum.

We further calculated the proportion of true negatives according to the test above (scenario A in Fig. 15a) from the subset of encounters without a GTSQ label for sepsis (negative class agreement) as well as the proportion of true positives (scenario B) from all encounters with a sepsis label (positive class agreement). The negative class percent agreement ranged from 89 to 94%, while the positive class percent agreement was only between 47 and 49% (Fig. 15b). This is in line with the low sensitivities and high specificities of the clinical criteria.

The higher number of complete encounters with on-admission sepsis (209) compared to incident sepsis (109) with a higher proportion of *severe sepsis* as first sepsis label in the former (71.7% vs. 51.4%) led us to reason that the diagnosis of sepsis was a lesser diagnostic dilemma on admission and that clinical criteria may have been more accurate in detecting on-admission than incident sepsis as labeled by experts. Compared to overall test performance, their sensitivities were indeed slightly increased to 59–66% for on-admission but reduced to 29–38% for incident sepsis while specificities remained similar (89–91% and 85–89%, respectively). Additional file 1: Fig. S2 enumerates the comparison results and statistical measures of agreement and test performance for on-admission and incident sepsis.

### Rater feedback

The conduct of this survey may have influenced communication among members of the care team and their evaluation of patients. Therefore, informal 5–10 min interviews were conducted to obtain feedback on the following two questions (Q1 and Q2) from six senior intensivists, four of which were raters in this study and two who have rated since.

Q1: *Did the GTSQ survey change your communication with other members of the care team, e.g., during visits and shift changes, and in which way?* One of six raters replied in the negative. Four reported more regular communication between raters during shift change on current evaluation of patient status and therapy, which was felt to benefit continuity and to aid focusing care. Three perceived the same benefits from more focused communication with colleagues during visits.

Q2: *Did the GTSQ survey influence patient evaluation by yourself and/or other members of the care team and clinical practice and in which way?* One rater replied in the negative, and five confirmed that the survey influenced their own patient evaluation and their treatment decisions. Four perceived the survey as an additional incentive for more stringent evaluation and thought over decisions.

The raters’ feedback suggests that the GTSQ survey was overall conducive to team communication and to clinical assessment and care.

## Discussion

In the GTSQ survey, senior attending physicians in our interdisciplinary surgical ICU documented daily their opinion on each patient’s condition by classification according to the clinical concepts of SIRS, sever sepsis and septic shock by labelling related interventions and outcomes.

### Representativeness of study population

Demographic characteristics, ICU length of stay and mortality rates of the ICU encounters (Table 2) were very similar to what has previously been reported for Western European ICUs overall [3, 54]. Likewise, thorax and abdomen as the most frequent focus localizations (Table 3) agree with the reported number one and two positions of lung and abdomen as sites of infection in sepsis patients treated in Western European ICUs [55–57]. The overall frequencies of lung, kidney and brain dysfunction labels (Fig. 6) match the organ dysfunction profile from an earlier cross-sectional survey in sepsis patients treated in German ICUs [55]. SOFA scores at ICU admission for encounters without *sepsis*, with *sepsis/severe sepsis* and with *septic shock* (Fig. 2) were very similar to the values for admissions categorized accordingly in the Intensive Care over Nations Audit [3]. We also observed the same 2-to-1 ratio of admissions with sepsis (present-on-admission sepsis) to incident sepsis cases as reported therein [3]. These similarities support the representativeness of our ICU cohort, despite the high proportion of neurosurgical patients.

### Validity of working diagnosis labels

External consistency (congruency with pathophysiology) of the working diagnosis labels was supported by the expected correlations with clinical parameters, particularly the overall higher illness severity for GTSQs with a sepsis label than with a non-sepsis label (Additional file 1: Fig. S1). CCI values as measure of comorbidity were higher for encounters in the sepsis than in the non-sepsis categories, and the encounter length increased from *neither SIRS nor sepsis* to *SIRS*, *sepsis*, *severe sepsis*, and *septic shock* (Table 2). Cumulative blood culture draws, bronchial lavages and antimicrobial use also increased from *neither SIRS nor sepsis* to *SIRS*, *sepsis*, *severe sepsis*, and *septic shock*. At the questionnaire level, the proportion of antimicrobial use in association with a *sepsis* label was threefold higher than with a *SIRS* label and increased further for *severe sepsis* and *septic shock* labels (Table 3). Together, this indicates that overall patients with a sepsis label were more severely ill than patients without a sepsis label and were treated for infection, which supports the validity of our working diagnosis labels.

### Validity of further GTSQ items

Correlations among responses to different GTSQ items that agree with clinical expectations support item validity. Accordingly, the prevalence for both positive focus localization and source control measure was more than fourfold higher in the presence of any sepsis compared to any non-sepsis label (Table 3). Likewise, macrocirculatory abnormalities were characteristic of *septic shock* and acute cardiac dysfunction (Fig. 11). Moreover, the continuous increase in infectious cause organ dysfunction labels from *SIRS* to *sepsis*, *severe sepsis* and *septic shock* (Fig. 8c) is consistent with increasing organ dysfunction associated with increasing sepsis severity. The larger proportion of infectious than non-infectious cause acute organ dysfunction labels coinciding with a positive focus localization label (Fig. 9) is also plausible. The overall dominance of lung and kidney among acute organ dysfunction labels was reflected similarly across individual focus localization categories except intracranial/meningeal, where unsurprisingly acute brain dysfunction was number one (Fig. 10). It also appears reasonable that the overall leading focus localizations, thoracic and abdominal, predominated in the acute lung and gastrointestinal dysfunction categories, respectively. In the assessment of the 24-h trend in the patients’ clinical picture, the rather unexpected associations of improvement and deterioration, respectively, with transition from *neither SIRS nor sepsis* to any form of sepsis and vice versa, as well as with transition within the sepsis spectrum from a less to a more severe form and vice versa, were indeed blow 0.6%. And as may be expected, patients with a *neither SIRS nor sepsis* label and with a *septic shock* label were, pro rata, ranked most frequently among the three least and the three most severely ill, respectively (Table 3). These correlations and, additionally, the informal feedback from our raters, which suggest that the labels actually reflect clinical reasoning, support overall validity of the GTSQ labels.

### Reliability of working diagnosis labels

In the evaluation of interrater reliability for selected GTSQ items, we found almost perfect agreement between raters for sepsis diagnoses. Therefore, the working diagnosis ratings can be considered a homogenous clinical judgement and suitable data source for further studies. The good interrater reliability of various items and consistency for observed pathophysiological and clinical patterns with expectations together corroborate the validity and reliability of the working diagnosis labels and the GTSQ overall.

### Suspicion of infection revealed as an unclear clinical concept

In the absence of timely and positive pathogen detection, the clinical diagnosis of sepsis pivotally rests on a suspected infection. Suspicion of infection, however, is associated with diverse symptoms, which are already common in critically illness, making this clinical assessment particularly challenging in these patients. Unsurprisingly, the editing rate for Item 4 was lower than for other items and interrater reliability only slight.

We counted 157 GTSQs with a non-sepsis working diagnosis label and a concurrent label for suspected infection (Table 3). Neither the presence of *SIRS* nor SOFA ≥ 2 with a concomitant suspicion of infection necessarily prompted the assignment of a sepsis label. A *SIRS* label was also frequently paired with a proxy for suspicion of infection, i.e., antimicrobial therapy and blood culture orders, also without prompting a sepsis label. Possibly, a suspected infection was not considered as the driver of SIRS or organ dysfunction in these cases, but other etiologies for these conditions were either known or suspected, and patients were thus not rated as septic. It thus appears that the coincidence of suspicion of infection with SIRS and SOFA ≥ 2 does not adequately capture the causal relationship between infection and systemic inflammation (Sepsis-1/2) and infection and organ dysfunction (Sepsis-3), respectively. Hence, the applicability of this clinical concept as a foundation for defining sepsis in the critically ill is flawed.

### Absence of a general illness severity hierarchy in the SIRS-sepsis-severe sepsis spectrum

An unexpected observation was the 3.3-fold higher mortality for encounters in the *SIRS* category compared to both *sepsis* and *severe sepsis* (Table 2). The observed 6.7-fold lower mortalities in both these sepsis categories compared to *septic shock* agrees with possible roles of sepsis and severe sepsis as intermediate states in patients that eventually die subsequent to suffering septic shock and multiple organ failure [14, 15]. Given higher values for CCI and encounter length, we had however expected to find higher mortality rates for *sepsis* and *severe sepsis* than for *SIRS* encounters in accordance with a SIRS-sepsis hierarchy [13]. Our subgroup analysis (Table 4) revealed that the strong association of *SIRS* compared to *sepsis* with mortality was characteristic to neurosurgical but not non-neurosurgical patients. This is compatible with evidence that non-traumatic intracerebral hemorrhage including subarachnoid hemorrhage commonly triggers a sympathetic stress response that is characterized by SIRS and associated with high morbidity and mortality [48, 58–60]. This mechanism would agree with the lower admission CCI values of the neurological *SIRS* encounters compared to each of the three sepsis categories. Likewise, the high proportion of neurosurgical referrals among the incident sepsis cases (51.4%) is consistent with CNS injury-induced immunodepression that makes infection a leading complication following traumatic brain injury, stroke, and spinal cord injury [47]. From this, it appears that the overrepresentation of neurosurgical referrals has the potential to influence our survey results, which we considered through a subgroup analysis mentioned where appropriate hereafter.

The following unexpected correlations cast further doubt on a hierarchy in severity of illness increasing from *SIRS* to *sepsis* and *severe sepsis*. First, SOFA scores were overall not different among these three working diagnosis categories at the encounter level (Table 2 and Fig. 2), and also between *SIRS* and *sepsis* as well as *severe sepsis* at the GTSQ level (Fig. 7b). The same was observed in our subgroup analysis except for higher SOFA scores with non-neurosurgical GTSQs carrying a *severe sepsis* label than a *SIRS* and a *sepsis* label (Fig. 13). Second, we counted equal proportions of GTSQs with any acute organ dysfunction label among all edited GTSQs with a *SIRS* and a *sepsis* label, which notably was dependent on the contribution of non-infectious cause organ dysfunction labels (Fig. 8). Third, the proportion of GTSQs with more than one acute organ dysfunction label approximately doubled from *sepsis* to *SIRS* (Fig. 7a), and the proportions with organ dysfunction labels were almost consistently higher in *SIRS* than in *sepsis* with the non-neurosurgical GTSQs (Fig. 14b). Fourth, 1.7- and 1.3-fold higher proportions of labels for macrocirculatory abnormalities among the GTSQs with a *SIRS* label compared to *sepsis* and *severe sepsis*, respectively, (Table 3) also support the absence of a SIRS-sepsis disease hierarchy. The subgroup analysis revealed similar differences for *SIRS* and *sepsis* in both referral groups and for *SIRS* and *severe sepsis* in the non-neurosurgical GTSQs. Last but not least, the proportions of GTSQs with a *SIRS* label and concurrent assignment to the three most severely ill was 3.7-times higher than with a *sepsis* label (Table 3). The subgroup analysis revealed similar differences in ranking for both referral groups.

Taken together, comparisons of ICU mortality, SOFA score, expert rating of acute organ dysfunction, macrocirculatory abnormalities, and daily ranking of illness severity strongly suggest that overall illness severity in *SIRS* exceeds *sepsis* and is rather on a par with *severe sepsis*.

### Ground truth labels provide limited support for clinical criteria for Sepsis-1/2 and Sepsis-3

Although plagued with uncertainty as discussed above, suspicion of infection is the common clinical criterion of the Sepsis-1/2 and Sepsis-3 consensus definitions [12, 18]. Its implementation in our EHR according to Seymour et al. [33] excluded 16.6% of all complete encounters with a sepsis label. In the remainder, clinical criteria for Sepsis-1/2 and Sepsis-3 identified essentially the same encounters as sepsis cases. Likewise, detection of sepsis onset through both consensus definitions was timely, overdue, and untimely each in essentially the same subsets of encounters. Given this very high concordance, discriminatory performance of Sepsis-1/2 and Sepsis-3 was basically determined by suspicion of infection. Both consensus definitions correctly identified ≥ 89% of the sepsis-free encounters yielding specificities around 92%. Yet, they showed only fair agreement with ground truth labels for detecting sepsis onset, mainly due to late or absent detection (false negatives) resulting in sensitivities around 55% (Fig. 15b).

For the purpose of (basic) research into the early recognition of sepsis, a temporal sepsis label should arguably be at least as timely as the clinical recognition of sepsis, and any delay should be obviated. Among the domains to measure usefulness of criteria to operationalize sepsis proposed by Angus et al. [61], the subdomain “concurrent validity” must in fact be given the same high priority for the purpose of research as of clinical care and not lower as suggested previously [62]. Against expert labels, Sepsis-1/2 and Sepsis-3 both fail in this subdomain. The discrepancy from expert labels shows that clinical criteria for the consensus definitions of sepsis are ill-suited for research into the early prediction of the condition that our senior intensivists recognized as “sepsis”.

### Limitations

Our ground truth labels reflect current, local clinical practice in a single surgical ICU. This may lead to an overestimation of the interrater reliability. The GTSQ survey may also inadvertently have influenced clinical practice, which in turn may have influenced the survey results. And we cannot exclude that the Sepsis-3 concept [18], introduced few months prior to the start of the survey, impacted the assignment of our working diagnosis labels.

Commonly used agreement metrics such as K_α_, Cohen’s κ and Scott’s π, exhibit chance correction bias [63]. We used K_α_ as a measure of agreement in our interrater reliability analysis and validation of Sepsis-1/2 and Sepsis-3 clinical criteria against ground truth labels, where it is prone to yield unfairly low scores. Therefore, we additionally provide contingency tables and percent agreement values where appropriate.

Our assessment of validity focused on the working diagnosis label. Future studies should also evaluate correlations between labels for organ and circulatory dysfunctions and the clinical markers and interventions for these conditions.

In our incident sepsis cases, equal numbers of encounters transitioned from *neither SIRS nor sepsis* and from *SIRS* each to the three sepsis categories (Fig. 5). We previously described that algorithmic determination of changes in the numbers of SIRS criteria met in the 24 h before the diagnosis detected sepsis in a polytrauma cohort [64]. However, 24-h rating intervals are too long to capture such rapid changes in the working diagnosis status. Although daily ratings anchor the working diagnosis status in time, future ground truthing should aim at a closer-meshed label assignment.

In this cross-sectional analysis we have not yet explored potential patterns and time dependencies in our ground truth labels as well as individual SIRS and SOFA criteria. When interpreting the unexpected high illness severity associated with the *SIRS* label compared to *sepsis* and *severe sepsis*, we hence have to consider that over half of the GTSQs with a *SIRS* label were from sepsis encounters (Fig. 3) and that, in these, *SIRS* labels were assigned before, after, and in-between sepsis labels (Fig. 4).

## Conclusions

A clear process understanding of sepsis and a gold standard diagnostic test are still lacking. Hence, defining the syndrome remains a challenge. Clinical criteria for Sepsis-1/2 and Sepsis-3 are commonly used for predictive modeling of sepsis in the critically ill, including by ML-based approaches, while agreement of these definitions with clinical reality remains unclear. Moreover, inclusion of clinical parameters that underlie SIRS and SOFA in the model lead to circular predictions instead of uncovering new relationships in the data. To address these limitations, we sought to capture the clinical reality in our ICU by daily questionnaire survey among clinical experts, referred to as ground truth, as a pertinent reference for sepsis. The GTSQ exhibits validity by consistency with current understanding of critical illness pathophysiology, including sepsis pathogenesis, and reliability by high inter-rater agreement of sepsis versus non-sepsis labels. Suspicion of infection, however, emerges as an unclear clinical concept. Unexpectedly, labels for *SIRS* identified patient time with higher and similar illness severity compared to *sepsis* and *severe sepsis*, respectively.

With that said, it is unsurprising that the clinical criteria for Sepsis-1/2 and Sepsis-3 identified the same patients as septic. They were much better in ruling out than ruling in sepsis. Their discriminatory performance against GTSQ labels essentially hinged on the suspicion of infection criterion and mainly suffered from low sensitivity. This flawed reliance on an unclear clinical concept pleads for further leveraging consistent expert knowledge of sepsis. In doing so, one must keep in mind that expert labels for sepsis do not imply certainty in knowing whether and when infection accounted for patients’ conditions. Therefore, we explicitly refer to this label as “working diagnosis”. Notwithstanding “the widely acknowledged uncertainties plaguing sepsis”, quoting [65] (p. 15), and the call to embrace them, we propose recollecting what is “currently *knowable*” [65] while providing critical care, i.e., the ground truth. The added measurement burden may partly be offset by improved communication within the care team and support of the decision-making process through the survey as indicated by our rater feedback. The impact of conducting the survey on clinical care needs further investigation.

The absence of a general SIRS-sepsis hierarchy in illness severity as well as almost perfect mutual agreement and limited accuracy in detecting sepsis onset by clinical criteria for Sepsis-1/2 and Sepsis-3 alike compared to expert labels challenge the validity of the consensus definitions in critical illness. In addition to patients that present with SIRS or an elevated SOFA score at baseline, such as in the ICU, we argue that expert labels should also be used as a reference to validate clinical criteria of consensus definitions for sepsis in other patient populations. Ground truthing sepsis is an indispensable step towards not only a satisfying and expedient definition of sepsis for a certain purpose but towards a genuine definition of the syndrome. We advocate collecting ground truth for sepsis to evaluate agreement with clinical criteria in different healthcare systems and settings and to eventually advance its understanding and early diagnosis and treatment.

## Supplementary Information

**Additional file 1: Appendix S1.** Questionnaire for the evaluation of SIRS and sepsis. **Appendix S2.** Agreement and performance of clinical criteria for Sepsis-1/2 and Sepsis-3 compared to GTSQ sepsis labels. **Text S1.** GTSQ construction and survey implementation. **Text S2.** Encounter definition. **Text S3.** Methods for evaluation of interrater agreement. **Text S4.** Features for SIRS and SOFA. **Text S5.** Supplementary results of interrater reliability study. **Table S1.** Contingency table for working diagnoses (Item 3) of interrater reliability study. **Table S2.** Krippendorff’s α values for questionnaire items of interrater reliability study. **Table S3.** Additional measures of agreement of questionnaire items in interrater reliability study. **Table S4.** GTSQs with labels for acute organ dysfunction (Item 9) by working diagnosis (Item 3). **Table S5.** Association of acute organ dysfunction (Item 9) with focus localization (Item 5). **Table S6.** Characteristics of complete encounters by working diagnosis (Item 3) in the subgroup analysis. **Table S7.** Responses to GTSQ items by working diagnosis label (Item 3) in the subgroup analysis. **Fig. S1.** Clinical characteristics for all edited GTSQs by working diagnosis (Item 3). Values of clinical characteristics in the 2 PM–2 PM-rating intervals for all 7.291 edited GTSQs (cf. Table 3 of the main text) were retrieved from the ICU’s PDMS. Mean values are displayed as box plots colored by working diagnosis (Item 3) as indicated in the legend.

## Data Availability

The datasets generated and analyzed during the current study are not publicly available due to patient privacy but are available from the corresponding author on reasonable request.

## Abbreviations

CCI: Charlson comorbidity index
EHR: Electronic health record
GTSQ: Ground Truth for Sepsis Questionnaire
HIS: Hospital information system
ICU: Intensive care unit
K_α_: Krippendorff’s α
ML: Machine learning
NPV: Negative predictive value
PDMS: Patient data management system
PPV: Positive predictive value
SIRS: Systemic inflammatory response syndrome
SOFA: Sequential organ failure assessment
