# Supplementary material for: Ground truth labels challenge the validity of sepsis consensus definitions in critical illness
Source: J Transl Med. 2022 Jan 15;20:27. doi: 10.1186/s12967-022-03228-7 (PMC8760797; doi:10.1186/s12967-022-03228-7)
Supplement: Supplementary file 1 — Additional file 1: Appendix S1. Questionnaire for the evaluation of SIRS and sepsis. Appendix S2. Agreement and performance of clinical criteria for Sepsis-1/2 and Sepsis-3 compared to GTSQ sepsis labels. Text S1. GTSQ construction and survey implementation. Text S2. Encounter definition. Text S3. Methods for evaluation of interrater agreement. Text S4. Features for SIRS and SOFA. Text S5. Supplementary results of interrater reliability study. Table S1. Contingency table for working diagnoses (Item 3) of interrater reliability study. Table S2. Krippendorff’s α values for questionnaire items of interrater reliability study. Table S3. Additional measures of agreement of questionnaire items in interrater reliability study. Table S4. GTSQs with labels for acute organ dysfunction (Item 9) by working diagnosis (Item 3). Table S5. Association of acute organ dysfunction (Item 9) with focus localization (Item 5). Table S6. Characteristics of complete encounters by working diagnosis (Item 3) in the subgroup analysis. Table S7. Responses to GTSQ items by working diagnosis label (Item 3) in the subgroup analysis. Fig. S1. Clinical characteristics for all edited GTSQs by working diagnosis (Item 3). Values of clinical characteristics in the 2 PM–2 PM-rating intervals for all 7.291 edited GTSQs (cf. Table 3 of the main text) were retrieved from the ICU’s PDMS. Mean values are displayed as box plots colored by working diagnosis (Item 3) as indicated in the legend. [file 12967_2022_3228_MOESM1_ESM.pdf]

Additional file 1

Table of contents

Appendix S1 Questionnaire for the evaluation of SIRS and sepsis

Appendix S2 Agreement and performance of clinical criteria for Sepsis-1/2 and Sepsis-3 compared to GTSQ sepsis labels

Text S1 GTSQ construction and survey implementation

Text S2 Encounter definition

Text S3 Methods for evaluation of interrater agreement

Text S4 Features for SIRS and SOFA

Text S5 Supplementary results of interrater reliability study

Table S1 Contingency table for working diagnoses (Item 3) of interrater reliability study

Table S2 Krippendorff's α values for questionnaire items of interrater reliability study

Table S3 Additional measures of agreement of questionnaire items in interrater reliability study

Table S4 GTSQs with labels for acute organ dysfunction (Item 9) by working diagnosis (Item 3)

Table S5 Association of acute organ dysfunction (Item 9) with focus localization (Item 5)

Table S6 Characteristics of complete encounters by working diagnosis (Item 3) in the subgroup analysis

Table S7 Responses to GTSQ items by working diagnosis label (Item 3) in the subgroup analysis

Fig. S1 Clinical characteristics for all edited GTSQs by working diagnosis (Item 3)

Fig. S2 Comparison of agreement and test performance for clinical criteria against GTSQ labels as reference class for on-admission and incident sepsis

Appendix S1. Questionnaire for the evaluation of SIRS and sepsis

Patient name:_________________________ Patient identifier code______________________

Ward: ________ Room number: __________ Date: _______ Attending physician: ________

Questionnaire for the evaluation of SIRS and sepsis

(referring to the preceding 24 hours, always 2 PM - 2 PM)

New admission within the last 24 hours? O no O yes

1. According to clinical judgement of disease severity in comparison to all other currently treated intensive care patients, the patient belongs to the group of

- o the three most severely ill
- o the three least severely ill
- o none of the above

2. How did the overall clinical picture of the patient develop during the preceding 24 hours?

   O improved O deteriorated O unchanged

Explanation: ____

3. Currently and in accordance with clinical evaluation the patient has the working diagnosis:

- o neither SIRS nor sepsis
- o SIRS
- o sepsis
- o severe Sepsis
- o septic Shock

4. Has there been a suspicion of infection in the patient within the last 24 hours?

- o no
- o yes   O persistent suspicion for more than 24 hours

  O suspicion newly arisen within the preceding 24 hours

  O first pronouncement of suspicion: O yesterday O today; time_______

  reasons for suspicion?

  ____

  O treatment with antibiotics was first discussed: O yesterday O today; hour_______

  (in person or by telephone)

  reasons for not initiating treatment with antibiotics?

  O germ judged as not requiring treatment/non-pathogenic

  O suspicion of contamination of sample

  O no organ dysfunction

  O __________________________

  O measures taken: O catheter change, prophylactic

  O order of PCT-testing on the following day

  O Other___________________________________

5. Does the patient have a focus of infection?

- o no
- o yes, but localization is unclear
- o yes, namely (please choose)

	suspected	confirmed
abdominal		
thoracic		
urogenital		
intracranial/meningeal		
bone/joint		
skin		
blood stream		
catheter		
endocarditis		

6. Has any measure for infectious source control been taken within the preceding 24 hours (apart from treatment with antibiotics)?

- o no
- o yes, namely O surgical ______________________________
  - O interventional ______________________________
  - O catheter change ______________________________

7. Does the patient have a macrocirculatory abnormality/vasomotor failure?

- o no
- o yes, namely O increased requirement of intravascular volume replacement
  - O capillary leak
  - O requirement of catecholamine therapy

8. Is there indication of microcirculatory dysfunction/disturbed tissue perfusion?

- o no
- o yes, namely O clinical suspicion

  O recapillarization time > 2s

  O hyperlactatemia (> 2 mmol/l)

  O central venous oxygen saturation (ScvO2) > 80%

9. Does the patient according to clinical judgement have an organ dysfunction?

- o no
- o yes, namely (please choose)

	newly arisen within the preceding 24 hours	acutely arisen more than 24 hours ago	chronic preexisting	cause of dysfunction?
kidney				O infectious<br>O non-infectious<br>O unclear
lung				O infectious<br>O non-infectious<br>O unclear
heart				O infectious<br>O non-infectious<br>O unclear
liver				O infectious<br>O non-infectious<br>O unclear
gastrointestinal				O infectious<br>O non-infectious<br>O unclear
coagulation system				O infectious<br>O non-infectious<br>O unclear
bone marrow				O infectious<br>O non-infectious<br>O unclear
brain				O infectious<br>O non-infectious<br>O unclear

10. How will the state of the patient presumably develop in the next 24 hours?

- o improve
- o deteriorate
- o stay the same

  reason____

Appendix S2 Agreement and performance of clinical criteria for Sepsis-1/2 and Sepsis-3 compared to GTSQ sepsis labels

		**Reference class: GTSQ** *Sepsis or severe sepsis* or *septic shock*	
		No	Yes
**Test class: clinical criteria for sepsis-1/2**	No	397	98
	Yes	27	216

Percent Agreement:	83.1%	Sensitivity:	68.8%, 95% CI: 63.4–73.9%
Krippendorff's α:	0.640	Specificity:	93.6%, 95% CI: 90.9–95.8%

		**Reference class: GTSQ** *Sepsis or severe sepsis* or *septic shock*	
		No	Yes
**Test class: clinical criteria for sepsis-3**	No	423	72
	Yes	49	194

Percent Agreement:	83.6%	Sensitivity:	72.9%, 95% CI: 67.2–78.2%
Krippendorff's α:	0.637	Specificity:	89.6%, 95% CI: 86.5–92.2%

		**Reference class: GTSQ** *Severe sepsis* or *septic shock*	
		No	Yes
**Test class: clinical criteria for sepsis-1/2**	No	397	103
	Yes	27	211

Percent Agreement:	82.4%	Sensitivity:	67.2%, 95% CI: 61.7–72.4%
Krippendorff's α:	0.624	Specificity:	93.6%, 95% CI: 90.9–95.8%

Appendix S2 Agreement and performance of clinical criteria for Sepsis-1/2 and Sepsis-3 compared to GTSQ sepsis labels

		Reference class: GTSQ *Severe sepsis* or *septic shock*	
		No	Yes
**Test class: clinical criteria for sepsis-3**	No	427	73
	Yes	45	193

Percent Agreement:	84.0%	Sensitivity:	72.6%, 95% CI: 66.8–77.8%
Krippendorff's α:	0.645	Specificity:	90.5%, 95% CI: 87.5–93.0%

Text S1

GTSQ construction and survey implementation

The first questionnaire draft was made by H.A.L and C.W. It featured items 3–6 and 9 which were extended by items 1, 2, 7, 8, and 10 during subsequent focus group discussions with four senior intensivists (J.K., D.M., T.F., T.K.) to warrant face and content validity of the measurement tool from the unanimous perspective of our raters. The GTSQ was piloted from 17/05/2016 to 18/07/2016 in a printed version. Focus group discussions were continued during this phase to achieve further disambiguation, practicality, and general applicability of the items. The final item order was adapted to reflect the clinical reasoning process. Group consensus was achieved for all decisions.

The electronic version of the GTSQ runs on a standard tablet computer's browser window and is completely self-contained. The implementation uses a combination of Hypertext Markup Language and JavaScript for the user interface and communicates with an underlying SQLite-database for data storage via PHP (footnote http://php.net). Network access on the tablet computer is permanently switched off for data security reasons.

The opening page featured a calendar date picker on month view with the current day highlighted and linked to the daily patient overview list incorporating item 1 on the next page. Selecting a name from the list opened a link to items 2–10 for this patient on a one-page scroll section.

During the survey period from 2 PM 18/07/2016 to 2 PM 08/07/2017, the patient list was updated daily between 7 AM and 2 PM according to current ICU PDMS census. Discharged patients were maintained in the list if their discharge occurred after the latest rating and were explicitly assigned "no bed". On average, 20.5 patients were listed daily. Rater-reported average daily GTSQ editing time was 45 minutes.

In response to unanimous rater feedback, the following three additions were made early in the survey period: On 01/08/2016, the list of infection foci in item 5 was extended by "endocarditis". On 29/09/2016, the distinction between "persistent" and "newly arisen" suspicion of infection was introduced in item 4, and classification of the cause of dysfunction for every organ as "infectious", "non-infectious", or "unclear" was introduced in item 9.

Our team of four senior intensivists (T.K., J.K., D.M., T.F.) was reduced to three (minus T.K.) at the end of November 2016 and extended to four again (plus S.N.) at the end of May 2017.

Text S2

Encounter definition

The time of PDMS admission and HIS discharge were defined as encounter start and end, respectively, with the following exceptions. The first vital sign marked the encounter start for 417 admissions out of 962 HIS-validated admissions because it was charted before PDMS admission (median difference = 0.37 h; range, 0.00–2.74 h). HIS discharge times were missing for 21 encounters, 18 of which thus ended at the last vital sign. The remaining 3 had no vital signs and <10 h between the defined encounter start and PDMS discharge which was defined as the respective encounter end. In 40 admissions, HIS discharge preceded the last vital sign (median difference = 0.4 h; range, 0.02–19.53 h) which hence marked the encounter end. One admission without vital sign was omitted because HIS discharge preceded PDMS admission by 20 minutes. In 16 of the thus defined encounters, a new encounter for the same patient started within ≤24 h after discharge (median = 11.92 h, range, 4.93–22.43 h). These adjoining encounters were concatenated.

Text S3

Methods for evaluation of interrater agreement

Sample size calculation

Given the high clinical expertise of all raters, we assumed a high proportion of agreement of 0.85 and as septic states were marginally equally distributed, we assumed an expected proportion of agreement of 0.55. Under the conditions that type I error=0.05, power=0.8, and K_0=0.5, we calculated a required sample size of 137 patients (Gao, 2012). A post-hoc power calculation based on the available 126 patients is compatible with these pre-specified assumptions except for requiring a minimally lower expected proportion of agreement of 0.54.

Measures of interrater reliability

To assess the degree of agreement between two or more raters we calculated kappa statistics. Because of the reported kappa paradox (Feinstein and Cicchetti, 1990) regarding the partly large effects of prevalence and rater bias on kappa values, we incorporated these issues into our analyses. For this, we assessed the marginal distributions, i.e., compared the rows and columns of the respective item's contingency table with the appropriate McNemar's or Bowker's test. This comparison was made for several items for all three pairings of intensivists separately. If marginal distributions varied greatly between raters, this would indicate a differing in their assessment of the frequency of the occurrence of a condition that is a bias in their decision making. Bias indices (Byrt et al., 1993) were computed as the difference in proportions for one condition in a binary setting. It ranges from 0 to 1, with 0 depicting no bias. Likewise, prevalence indices were calculated as the difference between the probabilities of 'Yes' and the probability of 'No', ranging between -1 and 1, with 0 illustrating equal prevalence of 50%. In case of high prevalence or index bias, we present the prevalence-adjusted bias-adjusted kappa (PABAK), as well as positive and negative agreement (Byrt et al., 1993, Cicchetti and Feinstein, 1990).

We assessed Krippendorff's α (K_α) for agreement, calculated with SAS macro Kalpha (Hayes and Krippendorff, 2007). K_α is suited for any number of raters and different scales of measurement and can handle missing values. In case of no rater bias present in the three

rater pairings, we calculated in a two rater setting, i.e., the tablet version of the questionnaire was rater 1 and the abridged paper-version of the questionnaire was rater 2. The five categorical sepsis diagnosis (*neither SIRS nor sepsis*, *SIRS*, *sepsis*, *severe sepsis*, *septic shock*) was considered nominally and ordinally.

References

Feinstein AR, Cicchetti DV. High agreement but low kappa: I. The problems of two paradoxes. J Clin Epidemiol. 1990;43(6):543-9.

Cicchetti DV, Feinstein AR. High agreement but low kappa: II. Resolving the paradoxes. J Clin Epidemiol. 1990;43(6):551–558.

Byrt T, Bishop J, Carlin JB. Bias, prevalence and kappa. J Clin Epidemiol. 1993 May;46(5):423-9.

Andrew F. Hayes & Klaus Krippendorff (2007) Answering the Call for a Standard Reliability Measure for Coding Data, Communication Methods and Measures, 1:1, 77-89

Fleiss, J. L. (1971). Measuring nominal scale agreement among many raters. Psychological Bulletin, 76(5), 378-382.

Gao 2012 www.mwsug.org/proceedings/2012/SA/MWSUG-2012-SA02.pdf

Text S4

Features for SIRS and SOFA

SIRS

The four SIRS criteria were determined based on the definitions in Bone et al. (1992). The temperature criterion was active if the body temperature is below 36 °C or above 38 °C, and the heart rate criterion was active if the heart rate was above 90 beats per minute. The respiration criterion was active if either the respiratory rate was above 20 breaths per minute or the partial pressure of carbon dioxide fell below 32 mmHg. Finally, leukocytes count were required to be below 4000 or above 12,000 per mm^3 for the last criterion to be active.

SOFA

The six SOFA dimensions were determined based on the definitions in Vincent et al. (1996). We followed exactly their thresholds to assign values between 0 and 4 to each of the SOFA dimensions for each time step. To generate timelines of each dimension's score we did not apply fixed length intervals but calculate the score at each measurement time of the corresponding feature. The dimension on the central nervous system, which is based on the Glasgow Coma Score (GCS), however could not be extracted from our PDMS system. We thus used the GCS-based SOFA-score assigned by two clinicians and co-authors F.S.C and J.J.S. for this dimension.

References

Bone RC, Balk RA, Cerra FB, Dellinger RP, Fein AM, Knaus WA, et al. Definitions for sepsis and organ failure and guidelines for the use of innovative therapies in sepsis. The ACCP/SCCM Consensus Conference Committee. American College of Chest Physicians/Society of Critical Care Medicine. Chest. 1992;101(6):1644-55. Epub 1992/06/01. doi: 10.1378/chest.101.6.1644. PubMed PMID: 1303622.

Vincent JL, Moreno R, Takala J, Willatts S, De Mendonca A, Bruining H, et al. The SOFA (Sepsis-related Organ Failure Assessment) score to describe organ dysfunction/failure. On behalf of the Working Group on Sepsis-Related Problems of the European Society of Intensive Care Medicine. Intensive Care Med. 1996;22(7):707-10. Epub 1996/07/01. doi: 10.1007/BF01709751. PubMed PMID: 8844239.

Text S5

Supplementary results of the interrater reliability study

The number of missings for the IRR study were very low, three patients had to be excluded for the analysis of suspected infection and 2 for the analysis of future development. All other items had no missings.

Working diagnoses (Item 3)

For binary distinctions between septic against non-septic conditions as well as for the five categorical working diagnosis variables, all bias indices for the three rater pairs were negligibly small. There were no significant differences in marginal homogeneities, neither for the aforementioned binary nor the five categorical variables. The marginal distributions indicated no prevalence problem in the binary settings, however, in the five categorical labels, the frequency of *sepsis* was lower than in the other categories (Supplementary File 6) Therefore, agreement measures were calculated in a two-rater setting (Supplementary File 8).

Suspected infection (Item 4)

There was a significant or borderline difference in marginal homogeneity between two rater pairs (p=0.025 and p=0.083, respectively) and bias indices ranged between 0.7 and 0.13. Moreover, the answer was ‘Yes‘ in only approximately 7% of the questionnaires and the prevalence indices for all three rater pairings were very high, ranging between 0.82–0.93. This makes interpretation of the low three rater setting K_α=0.18(-0.32–0.61) difficult and other measures such as the high PABAK of 0.79, the high observed proportion of agreement, 0.89, the expected proportion of agreement, 0.87, the high negative agreement, 0.94, and the low positive agreement of 0.24 should be considered additionally (Supplementary File 9).

Macrocirculation (Item 7)

Regarding the question on macrocirculatory abnormality/vasomotor failure, there was no indication of prevalence issues, and the K_α=0.77 (0.63–0.90) indicated substantial agreement. One pair of raters showed a significant bias, with McNemar’s p-value=0.025 and a bias index of 0.11. For overall agreement, we therefore also present additional measures in Supplementary File 9.

Acute organ dysfunction (Item 9)

The question concerning acute organ dysfunction was not affected by rater bias. However, kappas were influenced by the lower prevalence of ‘not having an organ dysfunction‘, as the majority of our patients had organ dysfunctions. Agreement was still substantial, K_α=0.68 (0.43–0.88), and correction for the prevalence issue gave a PABAK of 0.84. Observed and positive agreement where very high, 0.92 and 0.95, negative agreement was somewhat lower, 0.72.

Agreement for <u>specific organs</u> was very good for kidney, lung, heart and brain (see Supplementary File 8 for K_α). For these organs, there was no indication of rater bias or prevalence issues. However, both prevalence and bias issues were associated with liver, gastrointestinal, coagulation and bone marrow dysfunctions, as these specific organ dysfunctions were infrequent. The respective PABAKs were 0.87, 0.78, 0.84 and 0.84. Positive agreement was very high as expected, and negative agreement was lower at 0.69, 0.50, 0.64, and 0.64, respectively.

Future development (Expected 24-hour trend, Item 10)

The item evaluating future development was associated with major prevalence and significant bias problems. The by far most frequent answer was ‘no change‘ for all three raters, resulting in high prevalence indices. The agreement for the three categorical variable ‘deteriorate‘, ‘no change‘ and ‘improve‘ in the three rater setting was low, K_α=0.29 (0.11–0.45). The corresponding PABAK was 0.60.

Table S1 Contingency table for working diagnoses (Item 3) of interrater reliability study

		**Tablet questionnaire**				
		No SIRS or sepsis	SIRS	Sepsis	Severe sepsis	Septic shock
**Abridged paper version**	No SIRS or sepsis	40	5	1	0	0
	SIRS	0	17	0	1	0
	Sepsis	0	0	5	1	0
	Severe sepsis	2	0	1	14	2
	Septic shock	0	0	1	0	36

The three possible rater pairings were summarized in the absence of prevalence and bias problems.

Table S2 Krippendorff's α values for questionnaire items of interrater reliability study

Agreement measures for questionnaire items of interrater reliability study.

Item (number)	Krippendorff's α and 95% CIs
Sepsis diagnosis (3)	
Nominal, five categorical	0.85 (0.78-0.92)
Ordinal, five categorical	0.94 (0.90-0.97)
Binary	0.94 (0.86-1.0)
Suspected infection (4)	0.18 (-0.32-0.61)
Macrocirculatory abnormalities (7)	0.77 (0.63-0.90)
Acute organ dysfunction (9)	0.68 (0.43-0.88)
Kidney	0.81 (0.67-0.92)
Lung	0.70 (0.54-0.84)
Heart	0.85 (0.69-0.97)
Brain	0.75 (0.60-0.89)
Liver	0.66 (0.35-0.89)
Gastrointestinal	0.44 (0.14-0.70)
Coagulation	0.60 (0.29-0.85)
Bone Marrow	0.60 (0.29-0.85)
Expected 24-hour trend (10)	0.29 (0.11-0.45)

Table S3 Additional measures of agreement of questionnaire items in interrater reliability study

Item (number)	Prevalence Index	Bias Index	McNemar's test p-value	PABAK*	Observed proportion of agreement	Expected proportion of agreement	Positive agreement	Negative agreement
Suspected infection (4)	0.86	0.04	0.166	0.79	0.89	0.87	0.24	0.94
Macrocirculatory failure (7)	0.20	0	1	0.78	0.89	0.52	0.86	0.91
Acute organ dysfunction (9)	-0.71	0.03	0.206	0.84	0.92	0.76	0.95	0.72

* Prevalence-adjusted bias-adjusted kappa (Byrt et al., 1993).

Byrt T, Bishop J, Carlin JB. Bias, prevalence and kappa. J Clin Epidemiol. 1993 May;46(5):423-9.

Table S4 GTSQs with labels for acute organ dysfunction (Item 9) by working diagnosis (Item 3)

The table summarizes the response to item 9 for all GTSQs with ≥1 organ dysfunction label.

		All n=5793		Neither SIRS nor sepsis n=1727		SIRS n=842		Sepsis n=523		Severe sepsis n=1010		Septic shock n=1650	
Organ	Cause of dysfunction	N	(%)	N	(%)	N	(%)	N	(%)	N	(%)	N	(%)
Any organ	Infectious	2924	(50.5)	164	(9.50)	198	(23.5)	302	(57.7)	829	(82.1)	1431	(86.7)
Gastrointestinal	Infectious	513	(8.86)	20	(1.16)	17	(2.02)	35	(6.69)	118	(11.7)	323	(19.6)
	Non-infectious	180	(3.11)	15	(0.87)	48	(5.70)	20	(3.82)	20	(1.98)	77	(4.67)
	Unclear	30	(0.52)	4	(0.23)	1	(0.12)	6	(1.15)	19	(1.88)		
	Missing	108	(1.86)	2	(0.12)	2	(0.24)	8	(1.53)	19	(1.88)	77	(4.67)
Lung	Infectious	2451	(42.3)	114	(6.60)	153	(18.2)	208	(39.8)	704	(69.7)	1272	(77.1)
	Non-infectious	755	(13.0)	166	(9.61)	257	(30.5)	64	(12.2)	106	(10.5)	162	(9.82)
	Unclear	54	(0.93)	13	(0.75)	10	(1.19)	4	(0.76)	5	(0.50)	22	(1.33)
	Missing	701	(12.1)	55	(3.18)	91	(10.8)	96	(18.4)	133	(13.2)	326	(19.8)
Kidney	Infectious	1538	(26.5)	55	(3.18)	65	(7.72)	62	(11.9)	438	(43.4)	918	(55.6)
	Non-infectious	358	(6.18)	65	(3.76)	134	(15.9)	18	(3.44)	47	(4.65)	94	(5.70)
	Missing	370	(6.39)	10	(0.58)	37	(4.39)	29	(5.54)	82	(8.12)	212	(12.8)
Brain	Infectious	417	(7.20)	22	(1.27)	14	(1.66)	51	(9.75)	121	(12.0)	209	(12.7)
	Non-infectious	1330	(23.0)	600	(34.7)	271	(32.2)	99	(18.9)	78	(7.72)	282	(17.1)
	Unclear	35	(0.60)	2	(0.12)	6	(0.71)	8	(1.53)	3	(0.30)	16	(0.97)
	Missing	14	(0.24)	4	(0.23)	1	(0.12)	2	(0.38)	2	(0.20)	5	(0.30)
Heart	Infectious	629	(10.9)	5	(0.29)	5	(0.59)	27	(5.16)	90	(8.91)	502	(30.4)
	Non-infectious	282	(4.87)	49	(2.84)	113	(13.4)	21	(4.02)	15	(1.49)	84	(5.09)
	Unclear	16	(0.28)					4	(0.76)			12	(0.73)
	Missing	134	(2.31)	4	(0.23)	7	(0.83)	5	(0.96)	13	(1.29)	105	(6.36)
Coagulation system	Infectious	518	(8.94)	4	(0.23)	7	(0.83)	20	(3.82)	116	(11.5)	371	(22.5)
	Non-infectious	121	(2.09)	19	(1.10)	42	(4.99)	14	(2.68)	3	(0.30)	43	(2.61)
	Unclear	6	(0.10)			1	(0.12)	2	(0.38)			3	(0.18)
	Missing	85	(1.47)	6	(0.35)	1	(0.12)	2	(0.38)	5	(0.50)	71	(4.30)
Bone marrow	Infectious	654	(11.3)	10	(0.58)	23	(2.73)	28	(5.35)	147	(14.6)	446	(27.0)
	Non-infectious	154	(2.66)	17	(0.98)	59	(7.01)	13	(2.49)	30	(2.97)	35	(2.12)
	Unclear	3	(0.05)	1	(0.06)							2	(0.12)
	Missing	162	(2.80)	6	(0.35)	11	(1.31)	6	(1.15)	26	(2.57)	113	(6.85)
Liver	Infectious	478	(8.25)	4	(0.23)	2	(0.24)	9	(1.72)	129	(12.8)	334	(20.2)
	Non-infectious	164	(2.83)	3	(0.17)	53	(6.29)	5	(0.96)	21	(2.08)	82	(4.97)
	Unclear	25	(0.43)	3	(0.17)	5	(0.59)					17	(1.03)
	Missing	138	(2.38)	3	(0.17)	11	(1.31)	1	(0.19)	18	(1.78)	105	(6.36)

Table S5 Association of acute organ dysfunction (Item 9) with focus localization (Item 5)

Organ dysfunction = 100 percent

		None	Any	Unclear	Abdominal	Thoracic	Urogenital	Intracra-nial / meningeal	Joint / osseous	Cutaneous	Blood stream	Catheter associated	Endo-carditis
Organ	Total	N (%)	N (%)	N (%)	N (%)	N (%)	N (%)	N (%)	N (%)	N (%)	N (%)	N (%)	N (%)
None	1649	1313 (79.6)	334 (20.3)	41 (2.49)	78 (4.73)	95 (5.76)	14 (0.85)	49 (2.97)	23 (1.39)	40 (2.43)	30 (1.82)	7 (0.42)	3 (0.18)
Any	5267	1567 (29.8)	3700 (70.2)	108 (2.05)	1609 (30.5)	2073 (39.4)	134 (2.54)	219 (4.16)	370 (7.02)	241 (4.58)	233 (4.42)	30 (0.57)	8 (0.15)
Gastro-intestinal	831	88 (10.6)	743 (89.4)	12 (1.44)	598 (72.0)	291 (35.0)	7 (0.84)	17 (2.05)	33 (3.97)	30 (3.61)	61 (7.34)	7 (0.84)	1 (0.12)
Lung	3961	777 (19.6)	3184 (80.4)	67 (1.69)	1362 (34.4)	1985 (50.1)	117 (2.95)	120 (3.03)	305 (7.70)	197 (4.97)	197 (4.97)	27 (0.68)	6 (0.15)
Kidney	2266	351 (15.5)	1915 (84.5)	41 (1.81)	1037 (45.8)	1034 (45.6)	87 (3.84)	43 (1.90)	236 (10.4)	145 (6.40)	135 (5.96)	14 (0.62)	2 (0.09)
Brain	1796	852 (47.4)	944 (52.6)	53 (2.95)	328 (18.3)	482 (26.8)	8 (0.45)	149 (8.30)	98 (5.46)	100 (5.57)	78 (4.34)	15 (0.84)	4 (0.22)
Heart	1061	170 (16.0)	891 (84.0)	21 (1.98)	463 (43.6)	485 (45.7)	47 (4.43)	25 (2.36)	63 (5.94)	48 (4.52)	62 (5.84)	18 (1.70)	1 (0.09)
Coagulation system	730	79 (10.8)	651 (89.2)	18 (2.47)	367 (50.3)	356 (48.8)	35 (4.79)	9 (1.23)	91 (12.5)	91 (12.5)	29 (3.97)	9 (1.23)	1 (0.14)
Bone marrow	973	126 (12.9)	847 (87.1)	6 (0.62)	461 (47.4)	451 (46.4)	8 (0.82)	27 (2.77)	123 (12.6)	83 (8.53)	59 (6.06)	9 (0.92)	
Liver	805	91 (11.3)	714 (88.7)	10 (1.24)	429 (53.3)	375 (46.6)	17 (2.11)	24 (2.98)	117 (14.5)	75 (9.32)	30 (3.73)	7 (0.87)	

Focus localization = 100 percent

	None N=2982	Any N=4184	Unclear N=153	Abdominal N=1777	Thoracic N=2215	Urogenital N=159	Intracra-nial / meningeal N=274	Joint / osseous N=401	Cutaneous N=294	Blood stream N=273	Catheter associated N=37	Endo-carditis N=12
Organ	N (%)	N (%)	N (%)	N (%)	N (%)	N (%)	N (%)	N (%)	N (%)	N (%)	N (%)	N (%)
None	1313 (44.0)	334 (7.98)	41 (26.8)	78 (4.39)	95 (4.29)	14 (8.81)	49 (17.9)	23 (5.74)	40 (13.6)	30 (11.0)	7 (18.9)	3 (25.0)
Any	1567 (52.5)	3700 (88.4)	108 (70.6)	1609 (90.5)	2073 (93.6)	134 (84.3)	219 (79.9)	370 (92.3)	241 (82.0)	233 (85.3)	30 (81.1)	8 (66.7)
Gastro-intestinal	88 (2.95)	743 (17.8)	12 (7.84)	598 (33.7)	291 (13.1)	7 (4.40)	17 (6.20)	33 (8.23)	30 (10.2)	61 (22.3)	7 (18.9)	1 (8.33)
Lung	777 (26.1)	3184 (76.1)	67 (43.8)	1362 (76.6)	1985 (89.6)	117 (73.6)	120 (43.8)	305 (76.1)	197 (67.0)	197 (72.2)	27 (73.0)	6 (50.0)
Kidney	351 (11.8)	1915 (45.8)	41 (26.8)	1037 (58.4)	1034 (46.7)	87 (54.7)	43 (15.7)	236 (58.9)	145 (49.3)	135 (49.5)	14 (37.8)	2 (16.7)
Brain	852 (28.6)	944 (22.6)	53 (34.6)	328 (18.5)	482 (21.8)	8 (5.03)	149 (54.4)	98 (24.4)	100 (34.0)	78 (28.6)	15 (40.5)	4 (33.3)
Heart	170 (5.70)	891 (21.3)	21 (13.7)	463 (26.1)	485 (21.9)	47 (29.6)	25 (9.12)	63 (15.7)	48 (16.3)	62 (22.7)	18 (48.6)	1 (8.33)
Coagulation system	79 (2.65)	651 (15.6)	18 (11.8)	367 (20.7)	356 (16.1)	35 (22.0)	9 (3.28)	91 (22.7)	91 (31.0)	29 (10.6)	9 (24.3)	1 (8.33)
Bone marrow	126 (4.23)	847 (20.2)	6 (3.92)	461 (25.9)	451 (20.4)	8 (5.03)	27 (9.85)	123 (30.7)	83 (28.2)	59 (21.6)	9 (24.3)	
Liver	91 (3.05)	714 (17.1)	10 (6.54)	429 (24.1)	375 (16.9)	17 (10.7)	24 (8.76)	117 (29.2)	75 (25.5)	30 (11.0)	7 (18.9)	

Table S6 Characteristics of complete encounters by working diagnosis (Item 3) in the subgroup analysis

**Neurosurgical referrals**														
		All N=364		Neither SIRS nor sepsis N=215		SIRS N=57		Sepsis N=19		Severe sepsis N=15		Septic shock N=57	
		N	(%)	N	(%)	N	(%)	N	(%)	N	(%)	N	(%)
Men		195	(53.6)	107	(49.8)	30	(52.6)	16	(84.2)	9	(60.0)	33	(57.9)
Age group	< 40 yr	42	(11.5)	28	(13.0)	8	(14.0)			3	(20.0)	3	(5.26)
	40 - 60 yr	136	(37.4)	78	(36.3)	25	(43.9)	3	(15.8)	6	(40.0)	23	(40.4)
	> 60 yr	186	(51.1)	109	(50.7)	24	(42.1)	16	(84.2)	6	(40.0)	31	(54.4)
Working diagnosis on admission				211	(98.1)	24	(42.1)	5	(26.3)	6	(40.0)	13	(22.8)
Missing working diagnosis on day 1 or day 2				4	(1.86)	3	(5.26)	1	(5.26)			7	(12.3)
ICU mortality		60	(16.5)	16	(7.44)	17	(29.8)			1	(6.67)	26	(45.6)

Neurosurgical referrals							
		All<br>N=364	Neither SIRS nor sepsis<br>N=215	SIRS<br>N=57	Sepsis<br>N=19	Severe sepsis<br>N=15	Septic shock<br>N=57
Age	Mean (SD)	60.2 (15.7)	59.9 (15.6)	57.8 (17.1)	69.5 (10.4)	55.1 (20.3)	62.0 (14.0)
	Median (range)	61 (13–87)	61 (20–87)	58 (13–85)	72 (42–83)	58 (21–83)	63 (25–87)
Charlson comorbidity index	Mean (SD)	2.53 (2.72)	2.31 (2.67)	2.16 (2.21)	3.37 (3.20)	3.20 (2.60)	3.32 (3.05)
	Median (range)	2 (0–14)	2 (0–14)	2 (0–13)	3 (0–14)	3 (1–12)	3 (0–14)
Length of encounter, d	Mean (SD)	7.37 (8.57)	3.52 (3.90)	8.14 (5.92)	11.15 (8.26)	15.19 (9.40)	17.88 (12.12)
	Median (range)	4.09 (0.24–52.86)	1.85 (0.24–20.43)	6.87 (0.32–22.50)	10.01 (1.39–30.80)	12.64 (1.10–33.78)	16.19 (0.78–52.86)
Admission SOFA	Mean (SD)	5.48 (3.39)	4.11 (2.93)	6.98 (3.15)	7.05 (2.09)	6.60 (2.41)	8.45 (3.06)
	Median (range)	5 (0–16)	4 (0–13)	7 (0–16)	7 (4–10)	7 (2–10)	9 (1–14)
Maximum SOFA	Mean (SD)	6.44 (4.03)	4.44 (3.06)	8.19 (3.19)	8.26 (2.18)	8.00 (2.20)	11.26 (3.52)
	Median (range)	6 (0–22)	4 (0–15)	8 (0–16)	9 (4–11)	9 (5–11)	11 (4–22)
Antimicrobial therapy, ddd	Mean (SD)	5.36 (15.97)	0.32 (2.08)	1.36 (6.10)	8.21 (10.33)	19.84 (25.94)	23.70 (29.68)
	Median (range)	0.00 (0.00–181.33)	0.00 (0.00–23.38)	0.00 (0.00–45.00)	4.70 (0.00–40.82)	12.31 (0.00–99.14)	14.00 (0.00–181.33)

Neurosurgical referrals							
		All<br>N=364	Neither SIRS nor sepsis<br>N=215	SIRS<br>N=57	Sepsis<br>N=19	Severe sepsis<br>N=15	Septic shock<br>N=57
Microbiology testing – number of blood cultures	Mean (SD)	2.8 (4.2)	0.8 (1.5)	3.3 (2.6)	4.4 (3.9)	5.0 (3.6)	8.4 (6.3)
	Median (range)	1<br>(0–29)	0<br>(0–9)	2<br>(0–11)	3<br>(0–13)	5<br>(0–11)	7<br>(1–29)
Microbiology testing – number of Bronchiallavages	Mean (SD)	0.6 (1.5)	0.1 (0.4)	0.7 (1.4)	1.6 (1.5)	1.1 (1.3)	2.1 (2.5)
	Median (range)	0<br>(0–10)	0<br>(0–2)	0<br>(0–7)	1<br>(0–5)	1<br>(0–4)	1<br>(0–10)

Non-neurosurgical referrals													
		All N=392		Neither SIRS nor sepsis N=93		SIRS N=55		Sepsis N=32		Severe sepsis N=19		Septic shock N=190	
		N	(%)	N	(%)	N	(%)	N	(%)	N	(%)	N	(%)
Men		263	(67.1)	62	(66.7)	38	(69.1)	23	(71.9)	13	(68.4)	126	(66.3)
Age group	< 40 yr	38	(9.69)	10	(10.8)	8	(14.5)	3	(9.38)	2	(10.5)	15	(7.89)
	40 - 60 yr	113	(28.8)	20	(21.5)	15	(27.3)	10	(31.3)	7	(36.8)	61	(32.1)
	> 60 yr	241	(61.5)	63	(67.7)	32	(58.2)	19	(59.4)	10	(52.6)	114	(60.0)
Referring department	Anaesthesiology	42	(10.7)	1	(1.08)	1	(1.82)			2	(10.5)	38	(20.0)
	General surgery	168	(42.9)	19	(20.4)	29	(52.7)	13	(40.6)	6	(31.6)	100	(52.6)
	Gynaecology	9	(2.30)	5	(5.38)	2	(3.64)			1	(5.26)	1	(0.53)
	Internal medicine	12	(3.06)	6	(6.45)	1	(1.82)	1	(3.13)	1	(5.26)	3	(1.58)
	Neuroradiology	5	(1.28)	5	(5.38)								
	Orthopaedics and trauma centre	87	(22.2)	33	(35.5)	12	(21.8)	10	(31.3)	5	(26.3)	26	(13.7)
	Otorhinolaryngology	34	(8.67)	12	(12.9)	4	(7.27)	4	(12.5)	3	(15.8)	11	(5.79)
	Radiology	2	(0.51)									1	(0.53)
	Urology	27	(6.89)	10	(10.8)	6	(10.9)	4	(12.5)	1	(5.26)	6	(3.16)
	Other	8	(2.04)	4	(4.30)							4	(2.11)

Non-neurosurgical referrals							
		All N=392	Neither SIRS nor sepsis N=93	SIRS N=55	Sepsis N=32	Severe sepsis N=19	Septic shock N=190
		N (%)	N (%)	N (%)	N (%)	N (%)	N (%)
Referring department (encounters with more than one)	2	4 (1.02)	4 (4.30)				
Working diagnosis on admission			93 (100)	49 (89.1)	17 (53.1)	12 (63.2)	134 (70.5)
Missing working diagnosis on day 1 or day 2					2 (6.25)	1 (5.26)	7 (3.68)
ICU mortality		86 (21.9)	4 (4.30)	5 (9.09)	3 (9.38)	1 (5.26)	72 (37.9)

Non-neurosurgical referrals							
		All N=392	Neither SIRS nor sepsis N=93	SIRS N=55	Sepsis N=32	Severe sepsis N=19	Septic shock N=190
Age	Mean (SD)	63.6 (16.7)	65.7 (17.7)	63.1 (19.3)	63.5 (18.0)	59.1 (19.6)	63.0 (14.8)
	Median (range)	65 (7–94)	68 (8–93)	67 (19–94)	64.5 (24–89)	62 (7–83)	64.5 (14–94)
Charlson comorbidity index	Mean (SD)	3.30 (2.76)	3.29 (3.18)	2.91 (2.54)	3.22 (3.24)	3.63 (3.27)	3.37 (2.46)
	Median (range)	3 (0–13)	2.5 (0–13)	3 (0–10)	2 (0–11)	3 (0–11)	3 (0–13)
Length of encounter, d	Mean (SD)	10.30 (14.14)	2.08 (2.60)	3.09 (2.73)	8.24 (8.67)	9.09 (7.44)	17.04 (17.14)
	Median (range)	4.93 (0.05–104.85)	1.05 (0.15–18.70)	1.85 (0.37–13.68)	5.19 (0.57–33.67)	8.75 (0.35–22.76)	11.73 (0.05–104.85)
Admission SOFA	Mean (SD)	8.26 (4.34)	4.67 (2.80)	7.22 (3.51)	6.25 (2.74)	5.53 (2.93)	11.01 (3.62)
	Median (range)	8 (0–21)	5 (0–16)	7 (0–16)	6.5 (1–12)	6 (0–11)	11 (1–21)
Maximum SOFA	Mean (SD)	9.50 (5.02)	4.96 (2.89)	7.64 (3.54)	7.31 (2.78)	6.58 (3.25)	13.03 (3.97)
	Median (range)	9 (0–23)	5 (0–16)	8 (0–16)	7 (2–13)	6 (0–12)	13 (2–23)
Antimicrobial therapy, ddd	Mean (SD)	18.51 (39.69)	0.91 (2.97)	0.96 (3.64)	6.04 (8.84)	7.63 (7.58)	35.70 (51.48)
	Median (range)	4.52 (0.00–421.81)	0.00 (0.00–20.00)	0.00 (0.00–25.33)	4.56 (0.00–46.50)	5.69 (0.00–27.44)	18.65 (0.00–421.81)

Non-neurosurgical referrals							
		All N=392	Neither SIRS nor sepsis N=93	SIRS N=55	Sepsis N=32	Severe sepsis N=19	Septic shock N=190
Microbiology testing – number of blood cultures	Mean (SD)	4.8 (7.4)	0.6 (1.2)	1.3 (2.3)	4.4 (5.7)	5.7 (5.8)	7.9 (8.9)
	Median (range)	2 (0–48)	0 (0–7)	1 (0–15)	2 (0–23)	3 (0–21)	5 (0–48)
Microbiology testing – number of Bronchiallavages	Mean (SD)	1.6 (3.9)	0.1 (0.5)	0.4 (1.0)	0.5 (1.4)	1.3 (2.2)	2.9 (5.2)
	Median (range)	0 (0–50)	0 (0–3)	0 (0–6)	0 (0–6)	0 (0–9)	1 (0–50)

Table S7 Responses to GTSQ items by working diagnosis label (Item 3) in the subgroup analysis

**Neurosurgical referrals**						
	All edited GTSQs (n=2892)	Neither SIRS nor sepsis (n=1575)	SIRS (n=477)	Sepsis (n=234)	Severe sepsis (n=195)	Septic shock (n=359)
	N (%)	N (%)	N (%)	N (%)	N (%)	N (%)
Suspected infection	160/2511 (92.4)	49 (3.11)	51 (10.7)	16 (6.84)	14 (7.18)	30 (8.36)
Focus of infection (Yes/No)	929/1910 (98.2)	112 (7.11)	83 (17.4)	206 (88.0)	186 (95.4)	342 (95.3)
Focus localization unclear	74 (2.56)	39 (2.48)	16 (3.35)	6 (2.56)		13 (3.62)
Abdominal (suspected/confirmed)	43 (1.49)<br>136 (4.70)	 2 (0.13)	2 (0.42)<br>3 (0.63)	 22 (9.40)	5 (2.56)<br>53 (27.2)	36 (10.0)<br>56 (15.6)
Thoracic (suspected/confirmed)	251 (8.68)<br>216 (7.47)	20 (1.27)<br>7 (0.44)	23 (4.82)<br>22 (4.61)	41 (17.5)<br>65 (27.8)	43 (22.1)<br>33 (16.9)	124 (34.5)<br>89 (24.8)
Urogenital (suspected/confirmed)	8 (0.28)<br>5 (0.17)	2 (0.13)	2 (0.42)	3 (1.28)<br>5 (2.14)		1 (0.28)
Intracranial / meningeal (suspected/confirmed)	35 (1.21)<br>221 (7.64)	2 (0.13)<br>34 (2.16)	5 (1.05)<br>5 (1.05)	8 (3.42)<br>58 (24.8)	3 (1.54)<br>68 (34.9)	17 (4.74)<br>56 (15.6)
Joint / osseous (suspected/confirmed)	5 (0.17)<br>23 (0.80)		 6 (1.26)	 2 (0.85)	 5 (2.56)	5 (1.39)<br>10 (2.79)
Cutaneous (suspected/confirmed)	25 (0.86)<br>31 (1.07)	2 (0.13)<br>1 (0.06)		3 (1.28)<br>6 (2.56)	10 (5.13)<br>12 (6.15)	10 (2.79)<br>12 (3.34)
Blood stream (suspected/confirmed)	8 (0.28)<br>56 (1.94)	 2 (0.13)	 4 (0.84)	1 (0.43)<br>24 (10.3)	 10 (5.13)	7 (1.95)<br>16 (4.46)
Catheter associated (suspected/confirmed)	8 (0.28)<br>1 (0.03)	1 (0.06)<br>1 (0.06)		1 (0.43)		6 (1.67)
Endocarditis (suspected/confirmed)	4 (0.14)<br>6 (0.21)	1 (0.06)	1 (0.21)	1 (0.43)<br>3 (1.28)	1 (0.51)	 3 (0.84)

Neurosurgical referrals						
	All edited GTSQs (n=2892)	Neither SIRS nor sepsis (n=1575)	SIRS (n=477)	Sepsis (n=234)	Severe sepsis (n=195)	Septic shock (n=359)
	N (%)	N (%)	N (%)	N (%)	N (%)	N (%)
Macrocirculatory abnormalities	819/1982 (96.9)	182 (11.6)	171 (35.8)	58 (24.8)	80 (41.0)	328 (91.4)
Increased requirement of intravascular volume replacement	238 (8.23)	41 (2.60)	29 (6.08)	19 (8.12)	24 (12.3)	125 (34.8)
Capillary leak	75 (2.59)	2 (0.13)	4 (0.84)		2 (1.03)	67 (18.7)
Catecholamine requirement	780 (27.0)	171 (10.9)	167 (35.0)	51 (21.8)	68 (34.9)	323 (90.0)
Microcirculatory dysfunction	224/2580 (97.0)	32 (2.03)	34 (7.13)	6 (2.56)	14 (7.18)	138 (38.4)
Clinical suspicion	76 (2.63)		7 (1.47)		6 (3.08)	63 (17.5)
Recapillarization time > 2 s	9 (0.31)		1 (0.21)		1 (0.51)	7 (1.95)
Hyperlactatemia (> 2 mmol/L)	170 (5.88)	27 (1.71)	25 (5.24)	5 (2.14)	8 (4.10)	105 (29.2)
ScvO2 > 80 %	53 (1.83)	11 (0.70)	9 (1.89)	1 (0.43)	2 (1.03)	30 (8.36)
Acute or new organ dysfunction	1690 (58.4)	659 (41.8)	326 (68.3)	181 (77.4)	183 (93.8)	341 (95.0)
New organ dysfunction	121 (4.18)	45 (2.86)	29 (6.08)	6 (2.56)	6 (3.08)	35 (9.75)
No organ dysfunction	1065 (36.8)	869 (55.2)	134 (28.1)	46 (19.7)	6 (3.08)	10 (2.79)
Gastrointestinal	105 (3.63)	7 (0.44)	14 (2.94)	13 (5.56)	24 (12.3)	47 (13.1)
Lung	879 (30.4)	128 (8.13)	175 (36.7)	126 (53.8)	139 (71.3)	311 (86.6)
Kidney	309 (10.7)	36 (2.29)	74 (15.5)	18 (7.69)	54 (27.7)	127 (35.4)
Brain	1126 (38.9)	577 (36.6)	202 (42.3)	104 (44.4)	71 (36.4)	172 (47.9)
Heart	192 (6.64)	27 (1.71)	55 (11.5)	9 (3.85)	18 (9.23)	83 (23.1)
Coagulation system	62 (2.14)	6 (0.38)	5 (1.05)	14 (5.98)	5 (2.56)	32 (8.91)

Neurosurgical referrals						
	All edited GTSQs (n=2892)	Neither SIRS nor sepsis (n=1575)	SIRS (n=477)	Sepsis (n=234)	Severe sepsis (n=195)	Septic shock (n=359)
	N (%)	N (%)	N (%)	N (%)	N (%)	N (%)
Bone marrow	116 (4.01)	14 (0.89)	24 (5.03)	14 (5.98)	3 (1.54)	61 (17.0)
Liver	72 (2.49)	1 (0.06)	15 (3.14)		2 (1.03)	54 (15.0)
Source control	86/2733 (97.5)	13 (0.83)		17 (7.26)	8 (4.10)	45 (12.5)
Surgical	67 (2.32)	9 (0.57)		11 (4.70)	8 (4.10)	36 (10.0)
Interventional	3 (0.10)					3 (0.84)
Catheter change	16 (0.55)	4 (0.25)		6 (2.56)	1 (0.51)	5 (1.39)
Others	5 (0.17)			1 (0.43)		4 (1.11)
Preceding 24-hour trend	2836 (98.1)	1571 (99.7)	476 (99.8)	234 (100)	195 (100)	359 (100)
Improved	455 (15.7)	286 (18.2)	76 (15.9)	25 (10.7)	34 (17.4)	34 (9.47)
Deteriorated	388 (13.4)	115 (7.30)	98 (20.5)	34 (14.5)	27 (13.8)	113 (31.5)
Unchanged	1993 (68.9)	1170 (74.3)	302 (63.3)	175 (74.8)	134 (68.7)	212 (59.1)
Expected 24-hour trend	2806 (97.0)	1564 (99.3)	468 (98.1)	231 (98.7)	189 (96.9)	354 (98.6)
Improved	348 (12.0)	207 (13.1)	44 (9.22)	29 (12.4)	26 (13.3)	42 (11.7)
Deteriorated	151 (5.22)	52 (3.30)	34 (7.13)	3 (1.28)	9 (4.62)	53 (14.8)
Unchanged	2307 (79.8)	1305 (82.9)	390 (81.8)	199 (85.0)	154 (79.0)	259 (72.1)
Among 3 most severely ill ICU patients	269 (9.30)	75 (4.76)	65 (13.6)	8 (3.42)	7 (3.59)	111 (30.9)
Among 3 least severely ill ICU patients	531 (18.4)	432 (27.4)	54 (11.3)	22 (9.40)	7 (3.59)	14 (3.90)
Antimicrobial therapy	930 (32.2)	140 (8.89)	87 (18.2)	184 (78.6)	180 (92.3)	316 (88.0)

Neurosurgical referrals						
	All edited GTSQs (n=2892)	Neither SIRS nor sepsis (n=1575)	SIRS (n=477)	Sepsis (n=234)	Severe sepsis (n=195)	Septic shock (n=359)
	N (%)	N (%)	N (%)	N (%)	N (%)	N (%)
Microbiology testing – blood cultures	647 (22.4)	289 (18.3)	126 (26.4)	50 (21.4)	48 (24.6)	128 (35.7)
Microbiology testing – bronchial lavage	142 (4.91)	38 (2.41)	28 (5.87)	16 (6.84)	10 (5.13)	47 (13.1)

Neurosurgical referrals													
		All edited GTSQs (n=2892)		Neither SIRS nor sepsis (n=1575)		SIRS (n=477)		Sepsis (n=234)		Severe sepsis (n=195)		Septic shock (n=359)	
		N		N		N		N		N		N	
SOFA score	Mean (SD)	2844	5.50 (3.62)	1559	4.35 (2.92)	465	6.15 (3.14)	227	5.69 (2.72)	190	5.48 (2.54)	352	9.54 (4.65)
	Median (range)	2844	5 (0–22)	1559	4 (0–15)	465	6 (0–16)	227	6 (0–17)	190	5 (1–12)	352	10 (0–22)

Fig. S1 Clinical characteristics for all edited GTSQs by working diagnosis (Item 3)

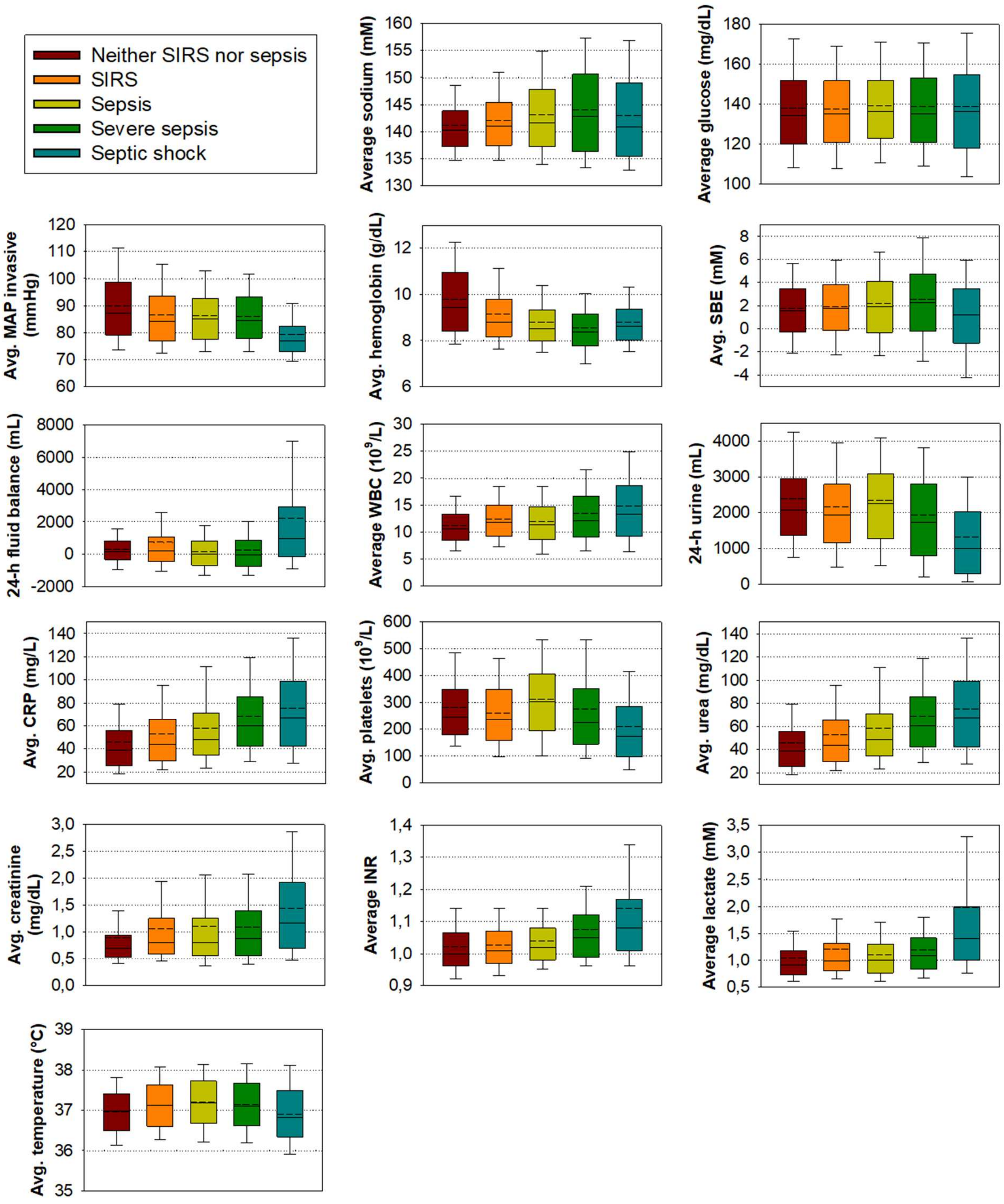

Values of clinical characteristics in the 2 PM–2 PM-rating intervals for all 7.291 edited GTSQs (cf. Table 3 of the main text) were retrieved from the ICU's PDMS. Mean values are displayed as box plots colored by working diagnosis (Item 3) as indicated in the legend.

Fig. S2 Comparison of agreement and test performance for clinical criteria against GTSQ labels as reference class for on-admission and incident sepsis

		**Consensus definition**			
**Clinical criteria**		**Sepsis-1/2** SIRS		**Sepsis-3** SOFA ≥2	
**GTSQ label**	*Sepsis*	●		●	
	Severe sepsis	●	●	●	●
	Septic shock	●	●	●	●
**On-admission sepsis**					
**Scenario** (agreement)	True negative	397	413	397	413
	True positive	113	108	115	110
	False negative	79	60	76	57
	False positive	40	48	41	49
**Agreement measures**	Percent agreement	0.811	0.828	0.814	0.831
	Krippendorf's α	0.525	0.551	0.535	0.561
**Test performance measures**	Sensitivity	0.589	0.643	0.602	0.659
	Specificity	0.908	0.896	0.906	0.894
	PPV	0.739	0.692	0.737	0.692
	NPV	0.834	0.873	0.839	0.879
**Incident sepsis**					
**Scenario** (agreement)	True negative	397	423	397	427
	True positive	33	20	28	17
	False negative	53	39	60	42
	False positive	50	73	48	69
**Agreement measures**	Percent agreement	0.807	0.798	0.797	0.800
	Krippendorf's α	0.276	0.141	0.222	0.110
**Test performance measures**	Sensitivity	0.384	0.339	0.318	0.288
	Specificity	0.888	0.853	0.892	0.861
	PPV	0.398	0.215	0.368	0.198
	NPV	0.882	0.916	0.869	0.910

GTSQ = Ground Truth for Sepsis Questionnaire, PPV = positive predictive value, NPV = negative predictive value
